# Recent Advances in Enzymatic and Non-Enzymatic Electrochemical Glucose Sensing

**DOI:** 10.3390/s21144672

**Published:** 2021-07-08

**Authors:** Mohamed H. Hassan, Cian Vyas, Bruce Grieve, Paulo Bartolo

**Affiliations:** 1 Department of Mechanical, Aerospace and Civil Engineering, University of Manchester, Manchester M13 9PL, UK; Mohamed.hassan@manchester.ac.uk (M.H.H.); Cian.Vyas@manchester.ac.uk (C.V.); 2 Department of Electrical & Electronic Engineering, University of Manchester, Manchester M13 9PL, UK; bruce.grieve@manchester.ac.uk

**Keywords:** enzymatic, non-enzymatic, glucose sensor, glucose oxidation, electrochemical sensor

## Abstract

The detection of glucose is crucial in the management of diabetes and other medical conditions but also crucial in a wide range of industries such as food and beverages. The development of glucose sensors in the past century has allowed diabetic patients to effectively manage their disease and has saved lives. First-generation glucose sensors have considerable limitations in sensitivity and selectivity which has spurred the development of more advanced approaches for both the medical and industrial sectors. The wide range of application areas has resulted in a range of materials and fabrication techniques to produce novel glucose sensors that have higher sensitivity and selectivity, lower cost, and are simpler to use. A major focus has been on the development of enzymatic electrochemical sensors, typically using glucose oxidase. However, non-enzymatic approaches using direct electrochemistry of glucose on noble metals are now a viable approach in glucose biosensor design. This review discusses the mechanisms of electrochemical glucose sensing with a focus on the different generations of enzymatic-based sensors, their recent advances, and provides an overview of the next generation of non-enzymatic sensors. Advancements in manufacturing techniques and materials are key in propelling the field of glucose sensing, however, significant limitations remain which are highlighted in this review and requires addressing to obtain a more stable, sensitive, selective, cost efficient, and real-time glucose sensor.

## 1. Introduction

Glucose detection is an important factor in the food, beverage, and fermentation manufacturing sectors as well as in the medical sector. The increased sugar consumption in people’s diet is related to many chronic health problems including cardiovascular diseases (including heart failure, stroke or heart attack), type 2 diabetes, sleep apnea, metabolic syndrome, and obesity [1,2]. Diabetes mellitus is a chronic condition that results in systemic and metabolic disorders. In 2019, diabetes affected 463 million people worldwide, was responsible for 1.5 million deaths, and the number of diabetic patients is expected to increase to 700 million by 2045 [3]. Moreover, diabetes is associated with other pathologies such as the risk of blindness, kidney failure, nerve damage, and heart problems [4]. Therefore, diabetic patients need to accurately determine their glucose blood level not just at the diagnosis stage but in all stages of treatment and disease management, and the use of non-invasive and rapid glucose level testing methods is critical [5,6,7,8]. Consequently, the food, beverage and fermentation manufacturing sectors are under pressure to reduce and control the level of sugar whilst maintaining the quality and safety of ingredients. This requires monitoring and measuring all analytes such as sugars, phenols, and alcohols throughout the manufacturing process and in the final product [2,9].

**Figure 1:**
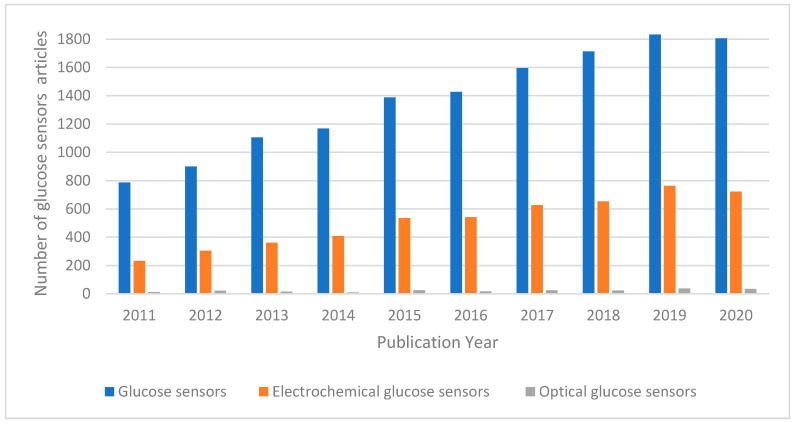
Number of glucose sensor related articles published in the past 10 years. The search was conducted using the Web of Science (Clarivate Analytics, Philadelphia, PA, USA) database considering the following keywords: “glucose”, “sensors”, “electrochemical” and “optical”.

In the last decade, the demand for glucose detection and monitoring systems significantly increased. This is reflected in the increased number of publications related to glucose sensors, illustrated in Figure 1. Glucose sensors comprise optical and electrochemical sensors. Optical glucose biosensors, encompassing different optical methods such as fluorescence, absorptiometry, and surface plasmon resonance (SPR) [6,10,11,12,13,14,15], use fibre optics to detect analytes using absorption, illumination, light scattering or refraction principles [16]. Optical biosensors have many advantages including remote sensing, low cost, electrical interference-free, and fast response time in comparison to normal test methods [17]. However, these sensors also present several disadvantages such as interference from surrounding light, the need for high-energy light sources, and limited concentration range, which limit their use [18].

**Figure 2:**
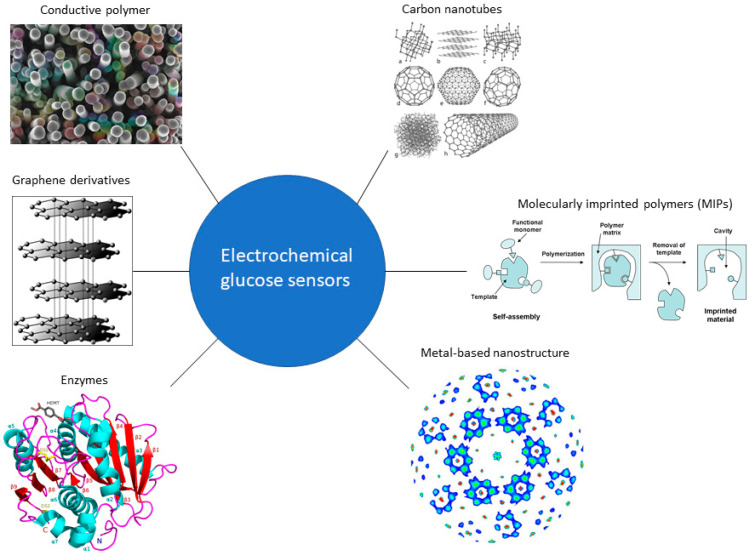
Materials used in electrochemical biosensors.

Electrochemical sensors, mainly based on amperometric methods, represent the most relevant group of glucose biosensors and comprise enzymatic and non-enzymatic sensors. Non-enzymatic amperometric glucose sensors are based on the direct electrochemical oxidation of glucose. A wide range of materials has been used in both enzymatic and non-enzymatic glucose biosensors (Figure 2) such as conductive polymers, enzymes, carbon nanotubes, and molecularly imprinted polymers (MIPs). MIPs mimic enzymes by creating polymeric crosslinked active sites for specific analytes, predominantly used in optical sensing they have been explored in recent years in electrochemical glucose sensing; however, they are out of the scope of this review [19,20,21]. Noble metals and their composites have been used specifically as the electrode materials for non-enzymatic sensors due to their high electrocatalytic activity, and high sensitivity to the electrooxidation of glucose [22,23,24,25]. The principle behind nonenzymatic glucose sensors was first reported by Walter Loeb [26] who observed a direct electro-oxidation of glucose in sulfuric acid-producing gluconic acid at a lead (Pb) anode. Direct electro-oxidation [27] and electroreduction [28] of glucose in alkaline (pH > 11) and acidic (pH < 2) solutions have also been investigated. The major problem faced by non-enzymatic glucose sensors is the absorption of glucose oxidation intermediates (e.g., CO) or solution active species (e.g., Cl^−^) which can lead to blockage of electrode activity for direct glucose electro-oxidation [29]. Furthermore, non-enzymatic amperometric glucose sensors suffer from a lower selectivity compared to enzymatic amperometric glucose biosensors due to the difficulty faced by the electrocatalytic materials to specifically catalyse glucose oxidation. However, non-enzymatic amperometric glucose sensors present long-term stability, which is the main drawback of the enzymatic biosensors due to the inherent nature of enzymes.

The principle behind enzymatic amperometric glucose sensors was proposed by Clark and Lyon in a patent describing the use of enzymes for converting electroinactive substrates into electroactive products [30]. Clark [31] also designed the first enzymatic amperometric glucose sensor by immobilising glucose oxidase (GOx) on a platinum (Pt) electrode. Since these preliminary studies, GOx has been extensively investigated and used for glucose biosensors due to its low cost, high bioactivity, selectivity, and stability [32]. Glucose dehydrogenase (GDH) is also used for blood glucose test strips [33,34,35,36]. GDH-based biosensors have the advantage of lower detection potentials compared to the first-generation of GOx-based biosensors, and their performance is independent of the oxygen level in the analyte solution [37]. Β-nicotinamide adenine dinucleotide (NAD)-dependent GDH and pyrroloquinoline quinone (PQQ)-dependent GDH (PQQGDH) are the two main types of GDH used in biosensor applications. However, GDH presents several limitations. PQQGDH suffers from low selectivity and requires suitable solubilisation detergents and purification to allow membrane binding, while water-soluble PQQGDH suffers from poor thermal stability [38]. NAD-dependent GDH biosensors require the addition of NAD cofactor which leads to complications (e.g., not always stable, contamination) in the analysis [37,39]. Furthermore, the electrochemistry of the oxidised form (NAD^+^) and reduced form (NADH) of NAD cofactor is irreversible. Direct oxidation of NADH at an unmodified electrode requires high overpotential due to its slow electron-transfer kinetics [37,38]. Moreover, for electrochemical measurements, GDH requires the use of artificial electron acceptors [40]. Alternatively, hexokinase isoenzyme II can be used for sugar sensing and glucose repression, as utilised by *Saccharomyes cerevisiao*, brewer’s yeast [41]. However, it is more expensive and presents lower stability when compared to GOx [38,40].

Glucose sensing has a significant scientific, clinical, and industrial relevance and significant progress has been made recently, particularly related to non-invasive methods to monitor blood [42,43,44,45]. Typically, glucose sensor reviews focus on a specific approach, enzymatic or non-enzymatic, in this review we discuss and compare both techniques and it is aimed at experts or new researchers in the field. This review discusses the key concepts of electrochemical glucose sensing, details the current state-of-the-art of non-enzymatic and GOx-based enzymatic glucose biosensors with a particular focus on materials and manufacturing technique, and presents the main research challenges and opportunities.

## 2. Three Generations of Enzymatic Glucose Sensors

The concept of a glucose enzyme electrode, as proposed by Clark and Lyon [30], monitors the oxygen consumption according to the following enzyme-catalysed reaction:

(1) $$\mathrm{glucose}+\mathrm{oxygen}\overset{\mathrm{GOx}}{\to }\mathrm{gluconic}\;\mathrm{acid}+\mathrm{hydrogen}\;\mathrm{peroxid}$$

The main problem faced by this sensor was the interference from background oxygen during the reaction. To solve this problem, Updike and Hicks [46] developed a system based on two oxygen working electrodes, measuring the current differential, hence, removing the noise created by the background oxygen. Similarly, Guilbault and Lubrano [47] developed an enzymatic amperometric glucose biosensor by monitoring the released hydrogen peroxide (H_2_O_2_) as follows:

(2) $$H_{2}O_{2}\to O_{2}+2H^{+}+2e^{-}$$

The catalytic reaction of GOx-based glucose biosensors involved the reduction of the enzyme’s flavin group (GOx(FAD)) to the reduced form (GOx(FADH_2_)) [48]:

(3) $$\mathrm{GOx}\left(\mathrm{FAD}\right)+\mathrm{glucose}\to \mathrm{GOx}\left({\mathrm{FADH}}_{2}\right)+\mathrm{gluconic}\;\mathrm{acid}$$

The reduction is then counteracted using an electron acceptor and oxidation mediator, (Med_ox_) to reoxidise the enzyme and regenerate the oxidised form (GOx(FAD)) [48]:

(4) $$\mathrm{GOx}\left({\mathrm{FADH}}_{2}\right)+{\mathrm{Med}}_{\mathrm{ox}}\to \mathrm{GOx}\left(\mathrm{FAD}\right)+{\mathrm{Med}}_{\mathrm{red}}$$

The regeneration of the enzyme is important to guarantee the enzymatic cycle, otherwise the enzyme will be reduced and cannot be reused, ending the sensing process.

**Figure 3:**
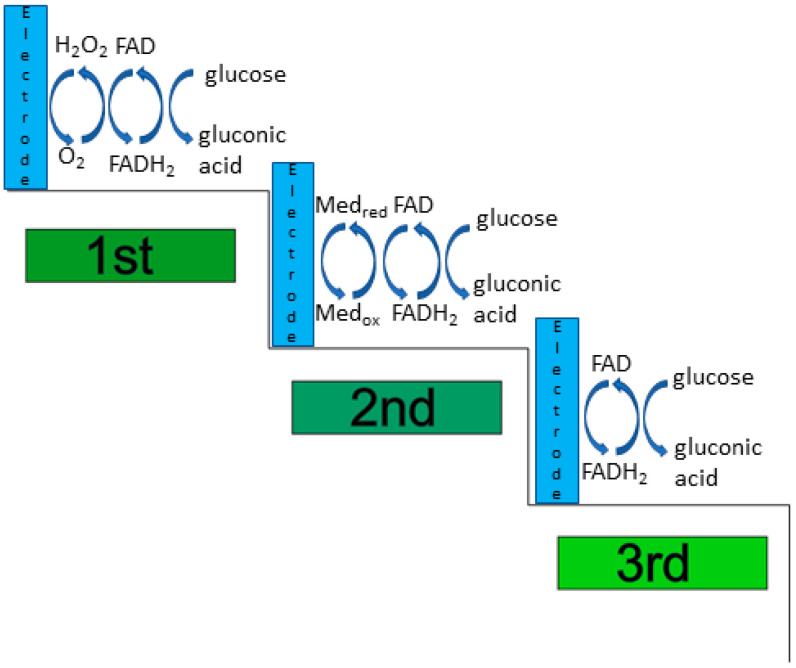
Schematic representation of enzymatic glucose oxidation mechanisms for the three different generations of biosensors.

According to the type of oxidation mediator, it is possible to identify three generations of glucose biosensors as shown in Figure 3. The first-generation of sensors use O_2_ as a physiological mediator, the second-generation uses an artificial (synthetic) electron acceptor, while the third-generation uses an electrode for direct electrical communication without requiring any mediators.

### 2.1. First-Generation Enzymatic Glucose Biosensors

The first generation of enzymatic glucose biosensors relied on oxygen as the oxidation mediator to regenerate GOx(FAD), thus detecting glucose by monitoring the oxygen consumption, or the generation of H_2_O_2_ during the enzymatic reaction [49]. The anodic oxidation and cathodic reduction of H_2_O_2_ were used to monitor the enzymatic generation process [50]. Moreover, the anodic oxidation of H_2_O_2_ enhances the ability to regenerate/replenish the oxygen, improving the enzymatic cycle [50]. The first-generation of enzymatic glucose biosensors were stable, simple, and easily used in miniaturised applications [51].

However, a major problem of the first-generation of glucose sensors was the electroactive interference as H_2_O_2_ requires a relatively high potential [51]. At a high potential level, some coexisting species such as ascorbic acid and uric acids are electroactive, reducing the selectivity and accuracy of the biosensor [52,53]. This problem was minimised by using a permselective membrane, reducing the access of the interferent to the surface of the biosensor transducer [54,55,56]. Different membrane materials such as Nafion (Nf), polycarbonate, cellulose acetate, poly(1,3-diaminobenzene), and polypyrrole (PPy) osmium (Os) complexes were used based on their transport properties, pore size, charge, or polarity [57,58,59,60]. Other approaches to minimise interference were based on the use of metallised carbon [61,62] and metal-hexacyanoferrate [49,63] transducers, to reduce the operational potential to the optimal potential region (0–0.2 V vs. Ag/AgCl) used for H_2_O_2_ detection thus avoiding the electroactivity of the interferents. Similarly, Wang and Wu [64], developed a glucose biosensor with high selectivity by dispersing rhodium particles in an Nf film.

An alternative approach is the use of Prussian blue (PB) to minimise interference, for example, Kafi et al. [65] fabricated a GOx/multiporous tinoxide nanofiber film on a PB-modified gold (Au) electrode for biosensing [65]. The aim was to develop a highly selective and low potential glucose biosensing, due to the PB having high catalytic activity and selectivity for the reduction of H_2_O_2_. Similarly, Li et al. [66] produced a highly sensitive imprinted electrochemical sensor based on double amplification using an inorganic PB catalytic polymer and GOx [66]. Cinti et al. synthesised PB nanoparticles on a filter paper, using it to immobilise GOx allowing the sensing of glucose in blood samples [67]. Moreover, a wide range of nanomaterials including carbon nanotubes (CNTs) [68], Pt nanoparticles [69], and composite nanomaterials [70,71] were successfully used to improve selectivity due to their high catalytic effect.

Another important limitation of the first generation of glucose biosensors, based on the use of oxygen as Med_ox_, was related to oxygen dependence [46,72]. These sensors were prone to errors, due to oxygen tension fluctuation and the stoichiometric limitation of oxygen, usually referred to “oxygen deficit” (the normal oxygen concentration is an order of magnitude lower than the physiological level of glucose) [73]. To overcome this problem, Gough et al. [74] developed a two-dimensional cylindrical electrode using a mass transport-limiting film to increase the O_2_/glucose permeability ratio. Other approaches included the use of an oxygen-rich carbon paste enzyme electrode [56,75,76] or an air diffusion biocathode that used oxygen directly from the air [77].

### 2.2. Second-Generation Enzymatic Glucose Biosensors

The second generation of enzymatic glucose biosensors relied on the use of an artificial Med_ox_ to mediate the GOx cycle instead of depending on oxygen as a mediator to transport electrons to and from the enzyme active site [78]. The artificial Med_ox_ can be an immobilised mediator directly attached to the enzyme or entrapped in an enzyme film [79,80], a solution-state mediator able to diffuse in and out of the enzyme active site [73], or a redox-conducting polymer able to transport its electrons to and from the enzyme active site [34,81,82]. Suitable mediators for GOx include conducting organic salts (particularly tetrathiafulvalene-tetracyanoquinodimethane, TTF-TCNQ), ferrocene, quinone compounds, ferricyanide, transition-metal complexes, phenothiazine, and phenoxazine compounds [51,83,84,85,86,87].

The catalytic process consisted of three steps: (1) the reduction of the GOx(FAD) to GOx(FADH_2_) due to the electron transfer from the glucose to the FAD reaction centres of GOx; (2) electrons transfer from the FADH_2_ centres to the artificial mediator (Med_ox_), hence reducing it from Med_ox_ to Med_red_; and (3) the transport of electrons through the artificial mediator to the electrode [73]. A current signal is produced due to the oxidation of Med_red_ and used for glucose measurement, which requires an efficient interaction between the enzymes and the mediators to guarantee the effective transportation of the electrons between the redox active centres and the electrode [79].

Several approaches have been proposed to tailor the mediators in the electrode-supported enzyme films, including using Os complex as a mediator, non-covalent functionalisation of multiwalled carbon nanotubes (MWCNTs), GOx and binding proteins, and stabilising artificial mediators [88,89,90]. Heller [90] was a pioneer in the field of directly connecting an enzyme redox centre to an electrode and the results of his work was a glucose biosensor with a sensitivity up to 1 A M^−1^ cm^−2^. Marquitan et al. [88] designed a redox polymer by crosslinking poly(4-styrene sulfonate-*co*-glycidyl methacrylate-*co*-butyl acrylate) (P(SS-GMA-BA)), and Os complex as a mediator producing a P(SS-GMA-BA)-Os matrix. GOx was then entrapped in the matrix forming a polymer/enzyme film used to modify the surface of a sub-micrometre scale carbon electrode [88]. Gallay et al. [91], designed a bienzymatic glucose biosensor based on the non-covalent functionalisation of MWCNTs with GOx and avidin (to allow the specific anchoring of biotinylated horseradish peroxidase (b-HRP)). Al-Sagur et al. [82] synthesised a multifunctional conducting polyacrylic acid (PAA) hydrogel (MFH) integrated with reduced graphene oxide (rGO), vinyl substituted polyaniline (VS-PANI), and lutetium phthalocyanine (LuPc2) to create a three dimensional (3D) robust matrix for GOx immobilisation (PAA-rGO/VS-PANI/LuPc2/GOx-MFH) and glucose measurement [82]. Schuhmann et al. [79] proposed the use of ferrocene amines directly attached to the surface of the enzyme through flexible linkages. Seketaryova et al. [73] designed a reagentless biosensor with free diffusing mediators by covalently bonding the GOx to the surface of the biosensor followed by exposing it to a water-organic mixture containing a high content of organic solvent [73]. In the case of immobilised mediator-based biosensors, it is important to immobilise the artificial mediator near both the enzyme’s redox centre and the electrode surface to ensure high electron-exchange efficiency. Contrary to solution-based mediator biosensors, the immobilised mediators suffer from limited range of motion.

### 2.3. Third-Generation Enzymatic Glucose Biosensors

The third generation of enzymatic glucose biosensors relies on direct energy transmission (DET), which depends on the distance between the enzyme’s redox centre and the electrode surface [92,93]. Several approaches were investigated to overcome the long electron tunnelling distance to achieve the direct electrochemistry of enzymes [94,95,96]. The reassembling of apo-proteins on cofactor modified enzymes and the reassembling of apo-enzymes on cofactor Au nanoparticles (AuNPs) are widely used strategies to align redox enzymes on the electrodes [94,95,97,98,99]. These methods are effective in the process of electrically wiring the redox enzyme to the electrode surface but are complex processes which limit their usage. The fundamental concept of DET was proposed by Heller and Degani [99] which demonstrated the possibility of connecting the enzyme active site covalently to the surface of the electrode using a redox polymer. Yehzekeli et al. [100] described a technique to electrically wire the enzyme, and the ability to transform the enzyme from an oxidase to hydrogenase by implanting Pt nanoclusters into GOx, which can be achieved by thermodynamically reducing Pt salts into Pt nanoclusters using the reduced factor FADH_2_. Several nanomaterials were described to directly achieve GOx electrochemistry [101,102,103,104]. Jose et al. [104] covalently immobilised GOx directly on the surface of MWCNT-coated electrospun Au fibre electrode. Tasviri et al. [102] produced an amine (NH_2_) functionalised tin-oxide (TiO_2_) coated carbon fibre nanotube (NH_2_–TiO_2_–CNT) layer used for the adsorption of GOx. The GOx containing matrix was then used to modify the surface of a glass carbon electrode (GCE) [102]. Zhang et al. [101] developed a glucose biosensor using bio-mediated AuNPs dispersed in a CNT–polyvinyl alcohol solution. After the dispersion, the GOx was added to the solution, which was then dried to produce a film. The obtained film was used to modify a GCE thus producing a mediator-free glucose biosensor. Holland et al. [105] achieved direct energy transfer between GOx and electrode via a site-specific modification of GOx to display a free thiol group near the active site, hence facilitating site-specific attachment of maleimide modified AuNPs to the enzyme. Tasca et al. [106] developed a cellobiose dehydrogenase (CDH) based glucose biosensor by directly adsorbing CDH to single-walled carbon nanotubes (SWCNTs). The CDH was extracted from Corynascus thermophilus (Ct) fungi and the results showed that the CtCDH can catalyse glucose oxidation in neutral pH. The biosensor was successfully used to detect glucose in both normal and diabetic patients under physiological conditions.

This third generation of glucose sensors produced better results than both the first and second generation, but still present restrictions stemming from their dependency on the enzyme’s activity which can be influenced by external environmental factors such as temperature, pH, and humidity [92,107,108]. Moreover, the biosensor performance also depends on the enzymatic layer thickness with high layer thickness resulting in signal dampening or loss [109,110].

Despite all these developments, the different generations of biosensors present several limitations not yet fully addressed, which has led to the development of non-enzymatic glucose detection systems. These non-enzymatic glucose sensors, sometimes referred to as the fourth generation of glucose sensors, rely on the concept of oxidising glucose directly on the electrode surface.

## 3. Recent Developments in Enzymatic Glucose Biosensors

**Figure 4:**
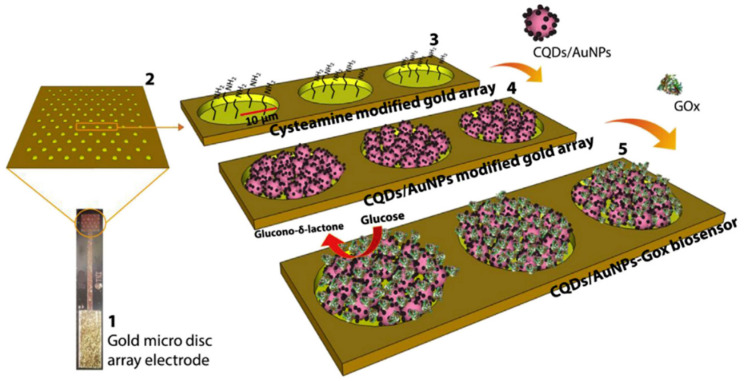
Preparation steps of a glucose biosensor using a micro disk array electrode. (1) single disk array electrode, (2) surface magnification, (3) cysteamine modification of the Au surface, (4) CQDs/AuNPs adhered surface, (5) GOx immobilisation to the surface [113].

Advances in the field of nanomaterials have led to the development of enzymatic biosensors incorporating nanomaterials (e.g., noble and transition metal nanoparticles, CNTs, graphene, and nanostructured metal oxides) to amplify the electron transfer rate, improving the biosensor performance in terms of selectivity and sensitivity [111]. Kumar-Krishnan et al. [112] developed a GOx immobilisation matrix by using AuNPs supported on a functionalised nano-silica (SiO_2_) surface using a deep eutectic solvent (DES). The SiO_2_ was first functionalised using a DES-mediated amine functionalisation to incorporate NH_2_ groups, and then the AuNPs were added to the solution to produce Au-SiO_2_NP. GOx was then covalently immobilised to the Au-SiO_2_NP followed by drop casting of the SiO_2_NP/GOx solution on the surface of a glass electrode. The obtained biosensor exhibited a sensitivity of 9.69 μAmM^−1^cm^−2^, and a wide linear range from 0.2 to 7 mM [112]. Buk and Pemble [113] prepared a glucose biosensor using a micro disk array electrode, modified with carbon quantum dots (CQDs)-AuNPs as a matrix for GOx (Figure 4). The microfabricated Au electrode was dipped in cysteamine to be functionalised by the NH_2_ groups, and then a solution containing CQDs mixed with AuNPs was drop casted on the surface of the electrode. Finally, glutaraldehyde was used to immobilise GOx, producing a micro disk array with a sensitivity of 626.06 μAmM^−1^cm^−2^ and a wide linear range from 0.16 to 4.32 mM [113].

MWCNTs were recently investigated to produce immobilisation matrices for GOx due to their high stability and ability for direct electron transfer [114,115,116]. Shrestha et al. [114] developed a bio-nanohybrid material by dispersing functionalised MWCNTs (fMWCNTs) in a Nf film dopped with PPy. GOx was then covalently immobilised in the material, producing Nf-GOx-fMWCNTs-PPy using electrochemical polymerisation on a surface of a Pt electrode [114]. The obtained Nf-GOx-fMWCNTs-PPy/Pt electrode detected glucose with a high sensitivity, 54.2 μA mM^−1^cm^−2^, in a linear range of up to 4.1 mM. Li et al. [115] used cobalt (II) sulphide nanoparticles (CoSNPs) to coat MWCNTs through an in situ hydrothermal method, obtaining a CoS-MWCNTs composite used as a matrix for GOx immobilisation. The CoS-MWCNTs was dispersed in water, GOx was added to the solution, and the mixture stirred gently to produce CoS-MWCNTS/GOx, which was drop casted to modify a GCE. Finally, to increase selectivity and sensitivity, Nf solution was added to the electrode surface resulting in the fabrication of a CoS-MWCNTs/GOx/GCE/Nf electrode with a sensitivity of 15 mA M^−1^cm^−2^ and a wide linear range from 8 µM to 1.5 mM [115]. Hao et al. [116] developed a functional nanocomposite by depositing manganese dioxide (MnO_2_) on the surface of MWCNTs via an in situ hydrothermal method. The nanocomposite (MnO_2_/MWCNTs) was used for the direct detection of H_2_O_2_, or as a matrix to immobilise GOx [116]. GOx was adsorbed by the MnO_2_/MWCNTs nanocomposite producing GOx/MnO_2_/MWCNTs, which was then used to modify the surface of a GCE producing an electrode with two distinct linear ranges from 5–200 μM and 0.2–1 mM [116].

Similarly, graphene has been used to produce enzymatic glucose biosensors. A disposable glucose biosensor was developed by Vukojevic et al. [117] using MnO_2_ nanoparticles to decorate graphene nanoribbons (GNR) followed by surface modification using a drop coating method with GOx and Nf. The MnO_2_-GNR composite solution was created using a simple hydrothermal mixing process. The composite solution was then drop cast on the screen printed carbon electrode (SPCE) producing a MnO_2_-GNR/SPCE electrode, followed by the addition of GOx and Nf, which resulted in an enzymatic glucose biosensor with a sensitivity of 56.32 μA mM^−1^cm^−2^ and a linear range from 0.1 to 1.4 mM [117]. Mao et al. [118] investigated the use of reduced graphene oxide (rGO) to increase the sensitivity and selectivity of a zinc oxide (ZnO) nanorod based biosensor. In this case, a polyethylene terephthalate (PET) substrate was used to hydrothermally synthetise the ZnO nanorods. Then, electrodeposited rGO was used to coat the ZnO/PET working electrode and AuNPs were dispersed on the surface leading to the production of ZnO/rGO/Au/PET. Finally, the GOx was physically adsorbed on the surface of the electrode leading to the fabrication of a GOx/Rgo/ZnO/Au/PET glucose biosensor with a sensitivity of 56.32 μA mM^−1^cm^−2^ and a linear range from 0.1 to 12 mM [118].

Recently, Hossain and Slaughter [119] proposed a hybrid glucose biosensor with high sensitivity and selectivity using both MWCNTs and graphene. Chemically derived graphene and MWCNTs functionalised with carboxylic groups were synthesised using a one-step solvothermal technique to produce a suspension containing both materials. This suspension was then drop casted on a Au electrode forming a thin film onto which PtNPs were electrochemically deposited. Finally, GOx was immobilised on the nanostructured electrode and coated with Nf. The fabricated hybrid biosensor exhibited sensitivity of 26.5 μA mM^−1^cm^−2^ and linear detection range from 0.5 to 13.5 mM [119].

High selectivity is a key requirement for glucose sensing applications. One approach to improve selectivity consists of using a red blood cell membrane (RBCM) as a diffusion barrier on the surface of the enzymatic glucose biosensor to eliminate any interfering molecules from reaching the surface [120]. The cell membrane’s main role is to block ions and small molecules to access the cell. However, the cell membrane contains glucose transporter-1 (GLUT1) proteins that promotes the exchange of glucose in and out of the cell [121,122]. As the RBCM is rich in GLUT1, if isolated from the cells, it can be used as a diffusion barrier on the surface of the enzymatic biosensor [123,124]. This approach was explored by Kim et al. [120] by coating a screen-printed Au electrode (SPGE) with glucose dehydrogenase (GDH), pyrroloquinoline (PQQ), mediator, and a buffer medium (0.1 M phosphate buffer solution). RBCM collected from red blood cells was used to coat the outer surface of the coated SPGE. The RBCM coated enzymatic glucose biosensor was then tested, showing lower limit of detection than the uncoated biosensor demonstrating that the RBCM increases the biosensor selectivity and its performance [120].

**Figure 5:**
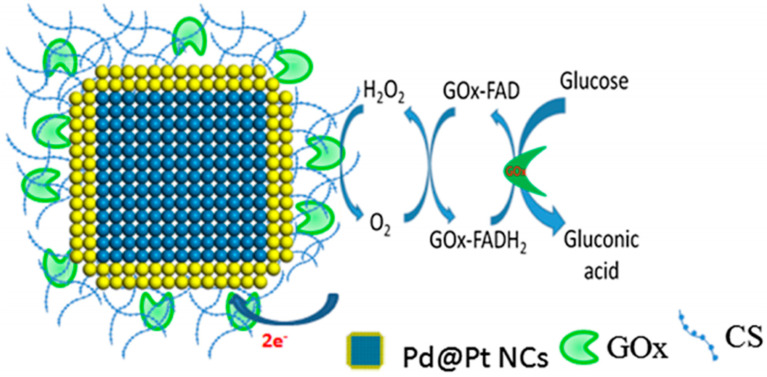
Schematic representation of the surface modification of Pb/Pt nanocubes using chitosan and covalent immobilisation of GOx [129].

Conductive polymers (CP), prepared mostly by incorporating conductive nanoparticles within a polymer matrix, can be used for enzyme immobilisation due to their unique properties such as high electron affinity, electrical conductivity, redox activity, stability, and low cost [125,126]. Soylemez et al. [127] fabricated an enzymatic glucose biosensor using a novel electrochromic conductive polymer, poly(2,5-di(furan-2-yl)thiazolo[5,4-d]thiazole) (PTTzFr), to immobilise GOx. PTTzFr, obtained using cyclic voltammetry (C-V), was drop coated by GOx using glutaraldehyde as a crosslinking agent. The biosensor exhibited a wide linear range from 5 μM to 0.7 mM. The biosensor readings were compared to readings from real sample analysis showing minor deviation [127]. Wang et al. [128] developed a ratiometric enzymatic glucose biosensor using schiff base polymers (SBPs) due to their stability, biocompatibility, and good mechanical and catalytic properties. Thionine, which carries two primary amine groups and p-benzaldehyde carrying aldehyde functional groups on the two sides of the ring, were the two monomers used to synthesise the SBPs. The SBPs nanosheets were used to immobilise GOx and the GOx/SBP matrix was used to modify a GCE via drop coating [128]. The biosensor showed two wide linear ranges from 1.97 μM to 4.0 mM at −0.2 V reference peak signal and from 0.82 μM to 4.0 mM at −0.05 V reference peak signal [128]. Krishnan et al. [129] designed Pb/Pt core/shell nano cubes electrode (Figure 5). The electrodes were coated with chitosan as an immobilisation matrix for GOx allowing the covalent bonding of the GOx to its surface via the active amine (NH) side group, improving stability and preserving the biocatalytic functions of the enzyme. The sensor showed a sensitivity of 6.82 μA mM^−1^cm^−2^ and a wide linear range from 1 to 6 mM [129].

**Figure 6:**
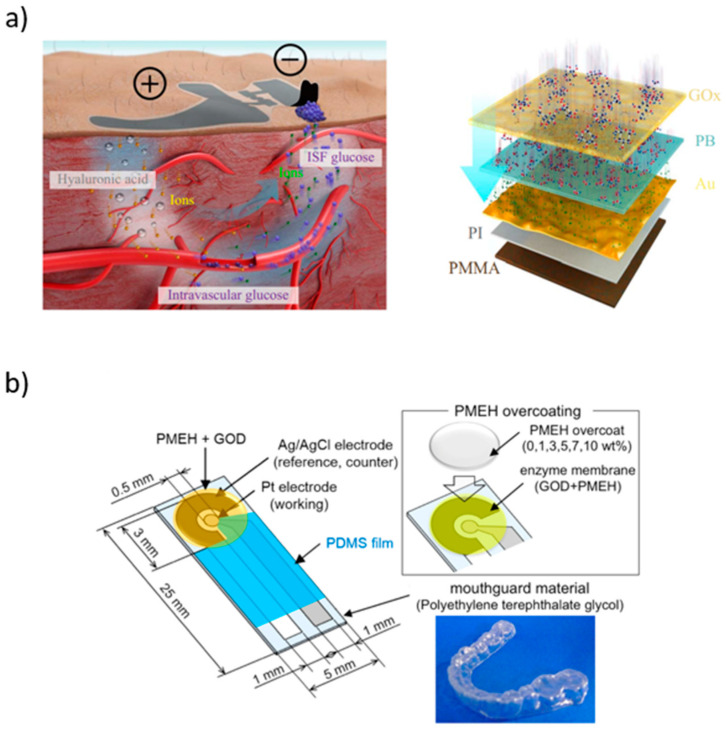
(**a**) Schematic representation of the ETC, which performs HA penetration, glucose refiltration, and glucose outward transportation (left image), and thin, flexible, and biocompatible paper battery attached to the skin surface for ETC measurement (right image) [131]. (**b**) Representation of the glucose biosensor on the PETG mouthguard support. Pt and Ag electrodes were formed on the PETG using a sputtering process. 30 units of GOx were applied to the sensing region of the working electrode [132].

Enzymatic glucose biosensors for blood glucose monitoring led to the development of patient friendly devices, enabling continuous and real-time glucose monitoring. Csoregi et al. [130] developed one of the earliest subcutaneous biosensors which led to the development of skin-like biosensors. This was a three-layered sensor based on a glucose sensing layer, a glucose mass transport restricting layer, and an outer biocompatible layer. The glucose sensing layer was prepared by cross-linking (poly[(l-vinylimidazolyl)osmium (4,4′-dimethylbipyridine)2Cl]}^+/2+^(PVI_13_-dme-Os) and GOX with poly(ethylene glycol) diglycidylether (PEG). The glucose mass transport restricting layer consisted of a poly(ester sulfonic acid) film (Eastman AQ 29D) and a copolymer of polyaziridine and poly(vinyl pyridine) partially quatemized with methylene carboxylate. Finally, the outer biocompatible layer was prepared by photo cross-linking tetraacylated poly(ethylene oxide). Below the glucose sensing layer, a flexible gold wire electrode was used. This layer was formed by sequentially depositing gold in a 0.09 mm deep shielded recess at the tip of a polyimide. The insulated 0.25 mm gold wire formed a “wired” glucose oxidase sensing layer. The produced 5 × 10^−4^ cm^−2^ sensor had a sensitivity range from 1 to 2.5 nA mM^−1^. An interesting example is the skin-like biosensor developed by Chen et al. [131] for non-invasive intravascular blood glucose monitoring (Figure 6a). The system compromises an ultra-thin (~3 μm) skin-like layer and a battery-powered paper. When the biosensor is attached to the skin it creates a subcutaneous electrochemical channel (ETC). Before implantation, the biosensor was sprayed with hyaluronic acid (HA). The ETC depends on the HA penetration into the interstitial fluid (ISF) (anode channel), intravascular blood glucose refiltration from vessels, and glucose reverse iontophoresis to the skin surface (cathode channel). The HA is repelled at the anode increasing the ISF osmotic pressure, forcing intervascular blood glucose to move out of the vessels towards the skin surface [131]. Arakawa et al. [132] developed a detachable mouth sensor to detect the level of glucose using saliva (Figure 6b). The biosensor is made of Pt and a silver/silver chloride (Ag/AgCl) electrode on a polyethylene terephthalate glycol (PETG) mouthguard, fitted with a wireless transmitter to enable direct readings. GOx is entrapped using a poly (2-methacryloyloxyethyl phosphorylcholine-co-2-ethylhexyl methacrylate (PMEH) film coating the Pt electrode, while the Ag/AgCl electrode is used as a reference electrode. Another relevant biosensor based on the detection of glucose in bodily fluid was proposed by Orzari et al. [133] which allowed the detection of glucose in sweat. In this case, a disposable biosensor was produced using graphite and a conductive ink. The conductive ink was printed on an adhesive sheet creating the electrode, followed by drop casting of GOx and dihexadecyl phosphate on the electrode surface. The biosensor could detect glucose in the sweat on the skin with a linear detection range of 1–10 µM.

## 4. Recent Developments in Non-Enzymatic Glucose Sensors

Non-enzymatic glucose sensing is a cheap and rapid technique that relies on the direct electrochemistry of glucose (oxidation or reduction) [28]. However, direct glucose oxidation on noble metal electrodes suffer from three major limitations [134,135,136,137,138]: (1) restricted glucose sensitivity which can be attributed to the slow glucose electro oxidative kinetics on conventional electrodes; (2) low selectivity as several sugars can be oxidised in the same potential range as glucose; and (3) reduced electrode activity due to ion contamination, mainly chloride ions (Cl^−^). The sensitivity and selectivity limitation can be countered by increasing the surface area of the electrode allowing more glucose to be in direct contact with the electrode’s surface. In order to achieve this, several nanomaterials are being investigated [139,140]. Particularly relevant are noble metals such as Pt, nickel (Ni), Ag, zinc, and Au, which are highly utilised to develop novel non-enzymatic glucose sensors [24,141,142,143]. The ion contamination limitation can be eliminated using alkaline conditions on the electrode surface, as the hydroxyl groups (OH) eliminates the chloride adsorption to the surface [29,144].

The non-enzymatic glucose oxidation catalytic process involves the hemiacetalic hydrogen atom abstraction that occurs in parallel with the adsorption of the organic species [145]. This process is regarded as the rate-determining step in the glucose electro-oxidation catalytic process. Bruke et al. [145] proposed the ‘‘incipient hydrous oxide adatom mediator’’ (IHOAM) model describing the complex electrocatalytic process of glucose. The IHOAM model describes the significance of the “active” hydroxide anions in the domain of the electrode surface produced by the separation of water:

(5) $$H_{2}O\to H^{+}+{\mathrm{OH}}^{-}$$

to the electro-oxidation of glucose and other organic compounds [137,138]. Moreover, the chemisorption of hydroxide anions to the reductive metal adsorption site (M), results in the production of oxidative adsorbed hydroxide radical (MOH_ads_) according to the following equation [146]:

(6) $$M+{\mathrm{OH}}^{-}\to {\mathrm{MOH}}_{\mathrm{ads}}$$

From Equations (5) and (6) it is possible to observe that the MOH_ads_ formation increases by increasing the concentration of OH^−^. Therefore, non-enzymatic glucose sensing is a pH dependent process, and an highly alkaline environment improves its sensitivity [144].

### 4.1. Metal-Based Glucose Sensors

Several metals, especially noble metals, have been studied as a base material for the electrodes of non-enzymatic glucose biosensors [28,147]. As a result, a deeper understanding of the glucose direct oxidation mechanism was achieved, showing that the mechanism depends directly on the metallic catalyst used in the electrode [110,137,138]. Moreover, advances in material science led to the development of several metal alloys and hybrid materials, allowing for improved properties when compared to noble metals and metal oxides alone [25,148,149,150].

#### 4.1.1. Pt-Based Glucose Sensors

**Figure 7:**
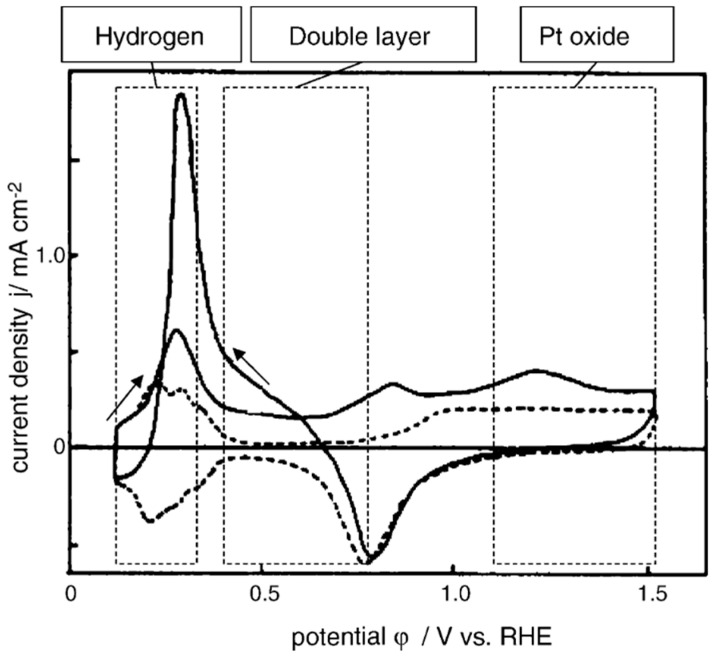
Cyclic voltammetry graph displaying three potential regions where glucose is electro-chemically oxidised at a Pt electrode [152].

Pt is widely used as an electrocatalytic electrode material due to its high catalytic activity and stability [145]. The glucose oxidation mechanism on the Pt electrode can be described using C-V, with the corresponding plots presenting three distinct peaks (Figure 7). The first peak (potential region 0.15–0.3 V vs. RHE (reversible hydrogen electrode)) corresponds to the hydrogen region and it is characterised by glucose dehydrogenisation leading to glucose adsorption to the electrode surface [145]. The second peak (potential region 0.4–0.8 V vs. RHE) represents the double layer region and it is associated to the water dissociation process (Equation (5)) followed by glucose oxidation that occurs at a lower potential than the required glucose thermodynamic oxidation potential as predicted by the IHOAM model [137,145]. The third region (potential region higher than 0.8 V vs. RHE), corresponds to the oxide region. In this region, the Pt electrode surface is oxidised changing to PtO. As a result, the glucose oxidation becomes diffusion-controlled, leading to direct bulk glucose oxidation on the oxide layer instead of a surface-bound reaction [137,151].

Noble metals such as Pt and Au, experience a large oxidative current in the double layer region during cathodic scan. Identical anodic currents appear during the cathodic scan for many other organic species, specifically alcohols [153]. Investigations of the produced oxidative currents have demonstrated the current dependence on the glucose concentration [154], pH [155], upper limit potential [144], surface morphology [156], and electrode ion contamination [27].

Strategies to overcome the Pt limitations comprise nanoengineering the Pt surface, fabricating nanocomposite structures, adjusting surface morphology, roughness, and increasing porosity [147,157,158,159,160]. Additionally, the fabrication of nanocomposite Pt-based structures is a widely used approach to improve the catalytic efficiency of noble metals [22,134,144]. This approach reduces production costs and the required amount of Pt and augments the surface catalytic activity by increasing the electrode surface area, evenly dispersing Pt on different substrates such as graphene [161], CNTs [134], and mesoporous carbon [162].

Xiao et al. [161] developed a flexible electrochemical glucose sensor using free-standing graphene paper carrying a nanocomposite PtAu alloy and MnO_2_. Electrodeposition was used to grow the PtAu-MnO_2_ nanocomposite on the graphene paper resulting in close contact between the PtAu alloy and MnO_2_. The glucose sensor exhibited high sensitivity of 58.54 μA mM^−1^cm^−2^ and a wide linear range from 0.1 mM to 30 mM [156,161,163]. Similarly, Hu et al. [156] developed a graphene-supported hollow Pt-Ni nanostructure electrode. The electrode was fabricated using a galvanic replacement approach at ambient temperature. The sensor presented high sensitivity of 30.3 μA mM^−1^cm^−2^ and a wide linear range from 0.5 mM to 20 mM. Chang et al. [163] used a hydrothermal synthesis approach to produce 22 nm Pt nanoclusters on GO using polyvinylproplidone as a surfactant. The produced sensor showed high sensitivity of 1.21 μA mM^−1^cm^−2^ and a wide linear range from 1 mM to 25 mM [163]. Nguyen et al. [134] developed a non-enzymatic biosensor by electrodepositing Au and Ruthenium (Ru) on the surface of a CNT-based Pt-nanoparticle hybrid composite in a poly(3,4-ethylenedioxythiophene) polystyrene sulfonate (PEDOT:PSS) conductive polymer. The sensor presented sensitivity of 0.234 μA mM^−1^cm^−2^ and a linear range of 10 mM [134].

A nano-porous Pt electrode is a 2D or a 3D porous film with nanosized pores usually produced using surface templated electrodeposition [164] and selective dealloying [165]. The pioneering work of Park et al. [166] demonstrated the potential of using nano porous Pt for non-enzymatic glucose sensing applications and this was followed by several other studies on Pt film electrodes [147,158,159]. Lee et al. [147], developed a prototype of a disposable non-enzymatic blood glucose sensing strip, using nano porous Pt as an electrode material mixed with poly(vinyl acetate) acting as a binding material. The mixture was then dispensed on the surface of a conducting circuit screen printed on a polyimide film. The sensor was able to detect glucose in whole human blood with acceptable stability for 30 days and a sensitivity of 0.0054 μAcm^−2^mgdL^−1^ [147]. Kim et al. [165] selectively dealloyed Si from Pt-Si alloy to create a nano porous Pt electrode with an increase in roughness due to the higher porosity which led to higher glucose sensitivity and lower sensitivity to interfering species such as ascorbic acid.

#### 4.1.2. Au-Based Glucose Sensors

Au is an extensively investigated electrode material, characterised by providing a high glucose oxidation current in both neutral and alkaline environments[164]. However, contrary to Pt its glucose oxidation mechanism is still vague, requiring further studies. In this case, the cyclic voltammetry graph only presents two regions, corresponding to the double layer and Au oxide region [26,29,105,155,167]. Previous studies seem to indicate a dependence between the oxidation mechanism and the presence of surface oxides such as Au(OH) [155]. Moreover, the glucose oxidation is not as dominant in the Au oxide region compared to Pt and mainly occurs in the double layer region where the surface OH_ads_ layers are formed [155]. Results also suggest that high pH levels result in higher faradic current, while at low pH the oxidative current is only detected at potentials higher than the oxide region [136,155,168].

Different methods such as electrochemical etching and dissolution [148,169,170], electrochemical deposition [171,172,173], and thermal annealing [174] have been used to produce nano porous Au samples aiming to reduce ion contamination and interference with the sensor surface. Verma [148], used Oryza sativa (Asian rice) extract as a reducing agent for the bio-reduction of Au (Au^3+^) and Ag (Ag^+^) ions, producing nano precursors leading to the formation of 0D monodispersed tunable nano porous AuNPs. The obtained nano porous AuNPs were then used to modify the surface of a GCE and tested for non-enzymatic glucose sensing using C-V. The electrode had a linear range from 1 to 50 μM and sensitivity of 6.67 μA μM^−1^cm^−2^ [148]. H_2_O_2_ detection can be also used as an indicator for the glucose presence in a sample. Xue et al. [169] used magnetron sputtering to fabricate nano porous Au thin films by chemically dealloying the nano porous Au to obtain a 3D bicontinuous ligament nanopore film. The film was then used to detect H_2_O_2_ with a linear relationship from 0.1 mM to 10 mM [169].

Sanzό et al. [173] used bubble electrodeposition to grow Au nanocorals on an Au screen-printed electrode. The modified electrode was used to directly detect glucose and was assessed using C-V, showing a linear range from 0.1 to 13 mM, and sensitivity of 0.5 μA mM^−1^cm^−2^ [173]. Han et al. [174] developed a new facile, environmentally friendly, cost-effective, and bottom-up approach to obtain a hierarchically porous Au cluster film for direct electrochemical non-enzymatic glucose sensing. The Au-cluster film consisted of a network structure interconnected with Au particles and disordered 3D hierarchical pores. The produced film showed a large surface area, high electrocatalysis, and electroconductivity towards glucose oxidation. The resulting film had a linear range from 0.01 to 10 mM and sensitivity of 10.76 μA μM^−1^cm^−2^ [174]. Scandurra et al. [175] used the dewetting technique to prepare a graphene paper-based electrode. In this case, an 8 nm thick Au layer was sputter deposited on a graphene paper and then a laser was used to dewet the Au layer. The laser-based dewetting resulted in smaller AuNPs on the electrode surface. The sensor had a sensitivity of 1240 μA mM^−1^ cm^−2^.

The major advantage of using Au-based electrodes for glucose sensing is the higher current response when compared to Pt-based electrodes, allowing for higher sensitivity and the ability to detect glucose in a neutral pH [155]. However, the main limitation of Au-based electrodes is related to the low glucose oxidation efficiency on the Au electrode surface, especially in the presence of surface OH_ads_ [138], which can be reduced by using arrays of nanoelectrodes spaced by non-electroactive materials. Additionally, as these electrodes are better activated in alkaline solutions they cannot be used for in-vivo studies, they suffer from surface contamination from anions such as phosphates and chlorides, and the selectivity is lower than Pt-based electrodes.

### 4.2. Transition Metals-Based Sensors

Pt and Au are suitable electrode materials for glucose detection but are expensive. Therefore, other non-precious transition metals [176,177] including Nickel (Ni) [27,141,178,179], and Copper (Cu) [180,181,182] and their oxides have been investigated. The redox reaction of transition metals does not follow IHOAM and chemisorption models. Under an anodic bias, transition metal’s oxide layer oxidizes into a higher oxidation number (i.e., Ni (II) to Ni (III)) [137,178,179]. The oxidative power of the higher oxide later has enough strength to create surface-bound OH_ads_ radicals, that oxidises organic compounds such as glucose on the electrode surface.

Previous studies highlighted that Cu (II) and Cu (III) couple on the anodic surface of the Cu electrode during glucose electro-oxidation in an alkaline environment [183,184]. Initially the Cu(OH)_2_ is oxidised to CuOOH, followed by hydrogen abstraction at the electrode surface, forming a radical intermediate and reforming the Cu(OH)_2_. Finally, the hydroxyl anions rapidly oxidise the radical intermediate producing gluconolactone [185]. The main disadvantage of a Cu electrode is its lack of ability to work in low or neutral pH as the CuOOH catalysis requires the presence of hydroxyl anions. Another important disadvantage is related to the competitive ethanol interference which negatively impacts the ability to detect blood glucose level.

The disadvantages associated with Cu were countered by using Cu nanostructured materials that enable an increase in the surface area. Recently Chen et al. [186], developed a portable micro glucose sensor using Cu oxide (CuO) nano-coral arrays (NCA) grown on a nano porous Cu (NPC) electrode. This non-enzymatic sensor showed high catalytic activity of glucose due to the CuO nano-coral arrays and high conductivity due to the NPC. The sensor had a sensitivity of 1621 μA mM^−1^ cm^−2^ and a linear range from 0.0005 to 5 mM. Zhang et al. [187] used a simple substrate-assisted electroless deposition technique to anchor Cu nanoparticles (CuNPs) on the surface of laser-induced graphene (LIG) producing a CuNPs-LIG composite sensor. The sensitivity of the sensor was 495 μA mM^−1^ cm^−2^, and the detection limit was 39 μM. Liu et al. [188] used a wet chemical technique combined with an annealing procedure to produce 3D copper oxide nanowire arrays (CuONWA) on a copper foam (CF) skeleton. The resulting CuONWA/CF platform was used as a glucose sensor, exhibiting a sensitivity of 32,330 μA mM^−1^ cm^−2^, and a linear range from 0.10 to 0.50 μM. The increased sensitivity of this sensor can be attributed to the increase in surface area due to the nanowire arrays as well as the porous copper foam.

In the case of Ni, which exhibits a similar glucose electro-oxidation mechanism to Cu, Ni (II) and Ni (III) couple mediate the glucose redox on the electrode surface. Gao et al. [189] used a femtosecond laser direct writing technique to prepare an Ni foam (NiF). The obtained NiF exhibited a controlled micro and nano superhydrophilicity structure leading to an increased detection area and higher sensitivity (13.822 mA mM^−1^ cm^−2^) and a detection limit of 0.0019 mM. Wang et al. [190] used a combination of solvothermal and ultrasonic techniques to synthesise a hierarchical 3D flower-like nickel (II)-terephthalic acid (Ni(TPA)) metal-organic framework (MOF), which was used to dope SWCNTs. The nanocomposite was then used to modify the surface of the GCE to detect glucose. The sensor had a linear range from 20 μM to 4.4 mM and a detection limit of 4.6 μM. Zhang et al. [191] developed a glucose-sensing composite through in situ self-assembly of Ni-based MOF in the presence of functional CNTs. The sensor had a sensitivity of 13.85 mA mM^−1^ cm^−2^, and linear detection range from 1 μM to 1.6 mM.

### 4.3. Metal Alloy-Based Glucose Sensors

Metal alloys are highly relevant electrode materials due to their high electrocatalysis. The different atoms inside an alloy create a new binding site or reaction pathway that can greatly impact the activation or binding energy of the reagent or intermediate, resulting in a possibly new reaction pathway and reduced overpotential [192]. Novel multi-metallic Pt and Au-based alloys and advances in computational chemistry have allowed development of electrodes with improved catalytic efficiency, stability, and anti-interference [25,149,150].

Several noble metal-based catalysts such as Pt-Ag [160], Pt-Ni [193], Pt-Pb [194], Pt-iridium (Pt-Ir) [195], and Au-Pt [149] were investigated for the development of novel electrodes using electrodeposition or selective dealloying techniques [150,185,196,197]. Lin et al. [160] prepared a PtAg/MWCNT modified GCE using drop casting. The sensor was used to determine the glucose level in bovine serum albumin samples and showed a linear detection range from 1 to 25 mM and sensitivity of 115.5 μA mM^−1^cm^−2^ [160]. The combined use of Pt and Ag resulted in a low overpotential, high sensitivity, and good stability when compared to monometallic-based non-enzymatic glucose sensors. Pt and Ag were also drop casted on the surface of a boron-doped diamond electrode (BDD) resulting in a modified electrode with high stability and selectivity [198]. Different percentages of Pt and Ag were investigated, and the best results were obtained for a 50/50 Pt to Ag ratio. The obtained non-enzymatic sensor had a linear detection range from 0.01 to 7.5 mM and a limit of detection of 0.007 mM [198].

Bimetallic systems were also investigated using one noble metal and one transition metal. Guascito et al. [196] modified the surface of a Pt electrode using tellurium microtubes through a drop casting method. The produced non-enzymatic glucose sensor was compared to a non-modified Pt electrode and showed higher sensitivity, stability, and reproducibility. The sensor exhibited two linear ranges with different sensitivity for each range-first range between 0.1 and 1 mM and sensitivity of 522.61 μA Mm^−1^cm^−2^ and second linear range from 1 to 29 mM with a sensitivity of 62.45 μA Mm^−1^cm^−2^ [196]. Guo et al. [199] fabricated Pt-Cu nanostrands (70/30 Pt to Cu ration) using wet chemistry and the nanostrands to modify the surface of a GCE. The produced sensor had a linear oxidation current of glucose ranging from 0.1 to 19 mM, sensitivity of 23 μA mM^−1^cm^−2^, and the interference from ascorbic acid, uric acid, and fructose was avoided [199].

Metal alloy-based non-enzymatic glucose sensors have great potential in facilitating glucose electro-oxidation with several studies [138] showing its higher sensing performance compared to monometallic-based sensors [200]. The alloy-based sensors typically based on Pt or Au are usually more expensive but present better current response and anti-interference, whilst operating in a neutral pH environment.

## 5. Conclusions and Future Perspectives

Due to health and regulatory pressures, the demand for low-cost, efficient, and accurate glucose sensors is significant, and the glucose market size is expected to be worth $36.7 billion by 2026 [201]. This paper reviews the current state-of-the-art of glucose sensors considering two major groups of sensors: enzymatic and non-enzymatic.

As discussed in detail, enzymatic-based glucose biosensors seem to correspond to the ideal model for glucose biosensing. However, many challenges such as short operational lifetime, temperature, and pH range, are limiting their performance requiring the use of more advanced materials and fabrication techniques. Currently, the most used materials are noble and transition metal nanoparticles, CNTs, graphene, and nanostructured metal oxides and different techniques were explored including using semi-permeable films, conductive polymers, and metal-based mediators. Further studies are also required to improve the selectivity and stability of these sensors and novel fabrication techniques should be considered to allow miniaturisation.

Besides glucose oxidase, other enzymes such as pyranose oxidase (POx) [202], which is a promising enzyme belonging to the glucose-methanol-choline (GMC) superfamily, have been investigated. POx has a higher thermal stability compared to GOx and it is not glycosylated thus presenting a shorter distance between the enzyme active site and the electrode, leading to better electron transfer and thus higher sensitivity. The main drawback of POx is its lower oxygen turnover compared to GOx which can interfere with its sensitivity. This drawback could be improved by tailoring the enzyme (e.g., genetic modification or selective breeding) [203,204].

Additive manufacturing (3D/4D printing) is developing at a fast rate with increasing choice of material selection [205]. Materials such as conductive polymers can be used in additive manufacturing to fabricate the enzymatic immobilisation matrix. Additive manufacturing builds products layer by layer which enables the fabrication of not only the surface of the biosensor but also a full biosensor with interchangeable materials to vary and control the biosensor properties. The combined use of additive manufacturing and smart materials (materials that respond to an external environmental stimulus), usually named as 4D printing [206,207], seems to offer significant potential to produce interactive systems that allow the biosensor to detect glucose only in specific scenarios.

Other potentially relevant manufacturing strategies, not fully explored, including screen-printed electrodes [208], ink jet sputtering [209], and nanolithography [210], all of them presenting advantages and drawbacks. Screen-printed electrodes are portable, disposable, low cost, and easy to use, but suffer from stability problems as the enzyme is immobilised in the ink which can leak with repetitive use, as well as ink dissolution [211]. Ink jet sputtering can be used to print any computer-generated pattern on the printing surface allowing the deposition of multilayers of enzymes, and the automatisation of the manufacturing process. However, this technique presents some limitations such as the lack of control on the layer homogeneity due to nozzle clogging and irregular droplet sizes, adverse effects on the enzymes due to printing stresses, and the potential contamination of the ink due to the refilling step [212]. Nanolithography allows for high fabrication resolution, being able to pattern biomolecular materials on many substrates, but the thermal force due to the increase in temperature can result in enzymatic denaturation and it is an expensive technique [213]. Moreover, the relationship between the enzyme’s conformation and the enzyme’s catalytic activity requires further research, which will potentially lead to significant innovations in the enzymatic biosensor field.

Contrary to enzymatic sensors, non-enzymatic glucose sensing presents higher stability, selectivity, less complex manufacturing procedures, and clinical uses [214]. The most used materials are Pt and Au composites and alloys, and popular techniques are electrodeposition, dewetting, and selective dealloying. Non-enzymatic sensors have been demonstrated to be functional for more than 30 days in undiluted whole blood after sterilisation, which is not possible with enzymatic sensors [214]. However, they still face several key challenges such as using alkaline operating conditions in testing, the high cost of materials, and the need for a protective layer on the surface to enhance selectivity.

Typically, glucose biosensor investigations focus on materials and manufacturing techniques, neglecting aspects related to the testing conditions. However, these testing conditions are usually impractical for usage in the human body which is the goal for clinical glucose sensors. A major limitation, which must be addressed in the future, is the lack of standards for testing and evaluation of different sensors against a specific criterion.

Further research is also required to fully understand the sensing mechanism of different materials and to explore techniques such as 3D/4D printing to produce glucose sensors, especially with the maturation and cost reduction in metal-based printing strategies such as electron beam melting [215]. Using different materials and combinations also hinders the commercialisation of non-enzymatic sensors which is a very important aspect to be considered. Disposable printed electrodes for direct electrochemical sensing would also help in reducing cost while increasing usage simplicity. The development of conducting polymeric nanowires, embedded metal nanoparticles in polymeric films, and polymeric ionic liquids are all important trends with high potential to expand the usage of biosensors in other fields such as point of care testing, agriculture, explosive and biological warfare agents detection [216].

Biosensors are a fast-growing field attracting researchers from different disciplines. Due to significant progress in areas related to advanced materials, manufacturing technologies, Internet of Things and artificial intelligence, it is foreseeable that the next generation (fifth generation) of biosensors will be able not only to monitor glucose levels but also able to respond to changing cues, predicting trends, and helping in managing glucose levels [217].

## Data Availability

Not applicable.

## References

[1] Ventura E.E., Davis J.N., Goran M.I. (2011). Sugar content of popular sweetened beverages based on objective laboratory analysis: Focus on fructose content. Obesity.

[2] Conzuelo F., Gamella M., Campuzano S., Ruiz M., Reviejo A., Pingarron J. (2010). An integrated amperometric biosensor for the determination of lactose in milk and dairy products. J. Agric. Food Chem..

[3] World Health Organization Leading Causes of Death Worldwide in 2019 (in Millions) 2020, Top 10 Causes of Death. https://www.who.int/news-room/fact-sheets/detail/the-top-10-causes-of-death#:~:text=The%20world%27s%20biggest%20killer%20is,8.9%20million%20deaths%20in%202019.

[4] World Health Organization (2009). Global Health Risks: Mortality and Burden of Disease Attributable to Selected Major Risks.

[5] Herman W.H. (2017). The global burden of diabetes: An overview. Diabetes Mellitus in Developing Countries and Underserved Communities.

[6] Bruen D., Delaney C., Florea L., Diamond D. (2017). Glucose Sensing for Diabetes Monitoring: Recent Developments. Sensors.

[7] Heller A., Feldman B. (2008). Electrochemical glucose sensors and their applications in diabetes management. Chem. Rev..

[8] Heller A. (2005). Integrated medical feedback systems for drug delivery. AIChE J..

[9] Kitikul J., Satienperakul S., Preechaworapun A., Pookmanee P., Tangkuaram T. (2017). A simple flow amperometric electrochemical biosensor based on chitosan scaffolds and gold nanowires modified on a glassy carbon electrode for detection of glutamate in food products. Electroanalysis.

[10] Ju J., Hsieh C.-M., Tian Y., Kang J., Chia R., Chang H., Bai Y., Xu C., Wang X., Liu Q. (2020). Surface Enhanced Raman Spectroscopy Based Biosensor with a Microneedle Array for Minimally Invasive In Vivo Glucose Measurements. ACS Sens..

[11] Bornhoeft L.R., Biswas A., McShane M.J. (2017). Composite hydrogels with engineered microdomains for optical glucose sensing at low oxygen conditions. Biosensors.

[12] Vafapour Z. (2019). Polarization-independent perfect optical metamaterial absorber as a glucose sensor in Food Industry applications. IEEE Trans. Nanobiosci..

[13] Shehab M., Ebrahim S., Soliman M. (2017). Graphene quantum dots prepared from glucose as optical sensor for glucose. J. Lumin..

[14] Jernelv I.L., Milenko K., Fuglerud S.S., Hjelme D.R., Ellingsen R., Aksnes A. (2019). A review of optical methods for continuous glucose monitoring. Appl. Spectrosc. Rev..

[15] Rachim V.P., Chung W.-Y. (2019). Wearable-band type visible-near infrared optical biosensor for non-invasive blood glucose monitoring. Sens. Actuators B Chem..

[16] Garzón V., Pinacho D.G., Bustos R.-H., Garzón G., Bustamante S. (2019). Optical Biosensors for Therapeutic Drug Monitoring. Biosensors.

[17] Bosch M.E., Sánchez A.J.R., Rojas F.S., Ojeda C.B. (2007). Recent development in optical fiber biosensors. Sensors.

[18] Kausar A.S.M.Z., Reza A.W., Latef T.A., Ullah M.H., Karim M.E. (2015). Optical nano antennas: State of the art, scope and challenges as a biosensor along with human exposure to nano-toxicology. Sensors.

[19] Ahmad O.S., Bedwell T.S., Esen C., Garcia-Cruz A., Piletsky S.A. (2019). Molecularly imprinted polymers in electrochemical and optical sensors. Trends Biotechnol..

[20] Garcia-Cruz A., Ahmad O., Alanazi K., Piletska E., Piletsky S. (2020). Generic sensor platform based on electro-responsive molecularly imprinted polymer nanoparticles (e-NanoMIPs). Microsyst. Nanoeng..

[21] Diouf A., Bouchikhi B., El Bari N. (2019). A nonenzymatic electrochemical glucose sensor based on molecularly imprinted polymer and its application in measuring saliva glucose. Mater. Sci. Eng. C.

[22] Deshmukh M.A., Kang B.-C., Ha T.-J. (2020). Non-enzymatic electrochemical glucose sensors based on polyaniline/reduced-graphene-oxide nanocomposites functionalized with silver nanoparticles. J. Mater. Chem. C.

[23] Wa Q., Xiong W., Zhao R., He Z., Chen Y., Wang X. (2019). Nanoscale Ni(OH)x films on carbon cloth prepared by atomic layer deposition and electrochemical activation for glucose sensing. ACS Appl. Nano Mater..

[24] Ma X., Tang K.-l., Yang M., Shi W., Zhao W. (2021). Metal–organic framework-derived yolk–shell hollow Ni/NiO@ C microspheres for bifunctional non-enzymatic glucose and hydrogen peroxide biosensors. J. Mater. Sci..

[25] Huo J., Lu L., Shen Z., Gao H., Liu H. (2020). Rational design of CoNi alloy and atomic Co/Ni composite as an efficient electrocatalyst. Surf. Innov..

[26] Casella I.G., Destradis A., Desimoni E. (1996). Colloidal gold supported onto glassy carbon substrates as an amperometric sensor for carbohydrates in flow injection and liquid chromatography. Analyst.

[27] Mohammed F.A., Khalaf M.M., Mohamed I.M., Saleh M., Abd El-Lateef H.M. (2020). Synthesis of mesoporous nickel ferrite nanoparticles by use of citrate framework methodology and application for electrooxidation of glucose in alkaline media. Microchem. J..

[28] Eryiğit M., Çepni E., Urhan B.K., Doğan H.Ö., Özer T.Ö. (2020). Nonenzymatic glucose sensor based on poly (3, 4-ethylene dioxythiophene)/electroreduced graphene oxide modified gold electrode. Synth. Met..

[29] Gao X., Du X., Liu D., Gao H., Wang P., Yang J. (2020). Core-shell gold-nickel nanostructures as highly selective and stable nonenzymatic glucose sensor for fermentation process. Sci. Rep..

[30] Clark L.C., Lyons C. (1962). Electrode systems for continuous monitoring in cardiovascular surgery. Ann. N. Y. Acad. Sci..

[31] Renneberg R., Pfeiffer D., Lisdat F., Wilson G., Wollenberger U., Ligler F., Turner A.P. (2007). Frieder Scheller and the short history of biosensors. Biosensing for the 21st Century.

[32] Bankar S.B., Bule M.V., Singhal R.S., Ananthanarayan L. (2009). Glucose oxidase—An overview. Biotechnol. Adv..

[33] Güneş M., Karakaya S., Dilgin Y. (2020). Development of an interference-minimized amperometric-FIA glucose biosensor at a pyrocatechol violet/glucose dehydrogenase-modified graphite pencil electrode. Chem. Pap..

[34] Cohen R., Cohen Y., Mukha D., Yehezkeli O. (2021). Oxygen insensitive amperometric glucose biosensor based on FAD dependent glucose dehydrogenase co-entrapped with DCPIP or DCNQ in a polydopamine layer. Electrochim. Acta.

[35] Iwasa H., Hiratsuka A., Tanaka T., Tsuji K., Kishimoto T., Watanabe Y., Hoshino Y., Muguruma H. (2020). Xylose-insensitive direct electron transfer biosensor strip with single-walled carbon nanotubes and novel fungal flavin adenine dinucleotide glucose dehydrogenase. IEEE Sens. J..

[36] Filipiak M.S., Vetter D., Thodkar K., Gutierrez-Sanz O., Jönsson-Niedziółka M., Tarasov A. (2020). Electron transfer from FAD-dependent glucose dehydrogenase to single-sheet graphene electrodes. Electrochim. Acta.

[37] Stolarczyk K., Rogalski J., Bilewicz R. (2020). NAD (P)-dependent glucose dehydrogenase: Applications for biosensors, bioelectrodes, and biofuel cells. Bioelectrochemistry.

[38] Lisdat F. (2020). PQQ-GDH–Structure, function and application in bioelectrochemistry. Bioelectrochemistry.

[39] Neethirajan S., Karunakaran C., Jayas D. Biosensors—An Emerging Technology for the Agricultural and Food Industry. Proceedings of the CSAE/SCGR 2005 Meeting.

[40] Ferri S., Kojima K., Sode K. (2011). Review of glucose oxidases and glucose dehydrogenases: A bird’s eye view of glucose sensing enzymes. J. Diabetes Sci. Technol..

[41] Kettner K., Krause U., Mosler S., Bodenstein C., Kriegel T.M., Rödel G. (2012). Saccharomyces cerevisiae gene YMR291W/TDA1 mediates the in vivo phosphorylation of hexokinase isoenzyme 2 at serine-15. FEBS Lett..

[42] Buford R.J., Green E.C., McClung M.J. A microwave frequency sensor for non-invasive blood-glucose measurement. Proceedings of the 2008 IEEE Sensors Applications Symposium.

[43] Omer A.E., Shaker G., Safavi-Naeini S., Kokabi H., Alquié G., Deshours F., Shubair R.M. (2020). Low-cost portable microwave sensor for non-invasive monitoring of blood glucose level: Novel design utilizing a four-cell CSRR hexagonal configuration. Sci. Rep..

[44] Karpova E.V., Karyakina E.E., Karyakin A.A. (2020). Wearable non-invasive monitors of diabetes and hypoxia through continuous analysis of sweat. Talanta.

[45] Alam M.M., Saha S., Saha P., Nur F.N., Moon N.N., Karim A., Azam S. D-care: A non-invasive glucose measuring technique for monitoring diabetes patients. Proceedings of the International Joint Conference on Computational Intelligence.

[46] Updike S., Hicks G. (1967). The enzyme electrode. Nature.

[47] Guilbault G., Lubrano G. (1973). An enzyme electrode for the amperometric determination of glucose. Anal. Chim. Acta.

[48] Chen C., Xie Q., Yang D., Xiao H., Fu Y., Tan Y., Yao S. (2013). Recent advances in electrochemical glucose biosensors: A review. RSC Adv..

[49] Karyakin A.A., Gitelmacher O.V., Karyakina E.E. (1995). Prussian blue-based first-generation biosensor. A sensitive amperometric electrode for glucose. Anal. Chem..

[50] Kaçar C., Dalkiran B., Erden P.E., Kiliç E. (2014). An amperometric hydrogen peroxide biosensor based on Co3O4 nanoparticles and multiwalled carbon nanotube modified glassy carbon electrode. Appl. Surf. Sci..

[51] Wang J. (2008). Electrochemical glucose biosensors. Chem. Rev..

[52] Poulos N.G., Hall J.R., Leopold M.C. (2015). Functional layer-by-layer design of xerogel-based first-generation amperometric glucose biosensors. Langmuir.

[53] Wang J. (2001). Glucose biosensors: 40 years of advances and challenges. Electroanalysis.

[54] Mǎdǎraş M.B., Buck R.P. (1996). Miniaturized biosensors employing electropolymerized permselective films and their use for creatinine assays in human serum. Anal. Chem..

[55] Palmisano F., Malitesta C., Centonze D., Zambonin P. (1995). Correlation between permselectivity and chemical structure of overoxidized polypyrrole membranes used in electroproduced enzyme biosensors. Anal. Chem..

[56] Jeerapan I., Sempionatto J.R., You J.-M., Wang J. (2018). Enzymatic glucose/oxygen biofuel cells: Use of oxygen-rich cathodes for operation under severe oxygen-deficit conditions. Biosens. Bioelectron..

[57] Sasso S.V., Pierce R.J., Walla R., Yacynych A.M. (1990). Electropolymerized 1, 2-diaminobenzene as a means to prevent interferences and fouling and to stabilize immobilized enzyme in electrochemical biosensors. Anal. Chem..

[58] Malitesta C., Palmisano F., Torsi L., Zambonin P.G. (1990). Glucose fast-response amperometric sensor based on glucose oxidase immobilized in an electropolymerized poly (o-phenylenediamine) film. Anal. Chem..

[59] Zhang Y., Hu Y., Wilson G.S., Moatti-Sirat D., Poitout V., Reach G. (1994). Elimination of the acetaminophen interference in an implantable glucose sensor. Anal. Chem..

[60] Emr S.A., Yacynych A.M. (1995). Use of polymer films in amperometric biosensors. Electroanalysis.

[61] Wang J., Liu J., Chen L., Lu F. (1994). Highly selective membrane-free, mediator-free glucose biosensor. Anal. Chem..

[62] Newman J.D., White S.F., Tothill I.E., Turner A.P. (1995). Catalytic materials, membranes, and fabrication technologies suitable for the construction of amperometric biosensors. Anal. Chem..

[63] Chi Q., Dong S. (1995). Amperometric biosensors based on the immobilization of oxidases in a Prussian blue film by electrochemical codeposition. Anal. Chim. Acta.

[64] Wang J., Wu H. (1995). Highly selective biosensing of glucose utilizing a glucose oxidase+ rhodium+ Nafion^®^ biocatalytic-electrocatalytic-permselective surface microstructure. J. Electroanal. Chem..

[65] Kafi A.K.M., Alim S., Jose R., Yusoff M.M. (2019). Fabrication of a glucose oxidase/multiporous tin-oxide nanofiber film on Prussian blue–modified gold electrode for biosensing. J. Electroanal. Chem..

[66] Melchels F.P., Domingos M.A., Klein T.J., Malda J., Bartolo P.J., Hutmacher D.W. (2012). Additive manufacturing of tissues and organs. Prog. Polym. Sci..

[67] Cinti S., Cusenza R., Moscone D., Arduini F. (2018). based synthesis of Prussian Blue Nanoparticles for the development of whole blood glucose electrochemical biosensor. Talanta.

[68] Alhans R., Singh A., Singhal C., Narang J., Wadhwa S., Mathur A. (2018). Comparative analysis of single-walled and multi-walled carbon nanotubes for electrochemical sensing of glucose on gold printed circuit boards. Mater. Sci. Eng. C.

[69] Tang H., Yan F., Lin P., Xu J., Chan H.L.W. (2011). Highly sensitive glucose biosensors based on organic electrochemical transistors using platinum gate electrodes modified with enzyme and nanomaterials. Adv. Funct. Mater..

[70] Baek S.H., Roh J., Park C.Y., Kim M.W., Shi R., Kailasa S.K., Park T.J. (2020). Cu-nanoflower decorated gold nanoparticles-graphene oxide nanofiber as electrochemical biosensor for glucose detection. Mater. Sci. Eng. C.

[71] Mei Q., Fu R., Ding Y., Wang A., Duan D., Ye D. (2019). Electrospinning of highly dispersed Ni/CoO carbon nanofiber and its application in glucose electrochemical sensor. J. Electroanal. Chem..

[72] Reach G., Wilson G.S. (1992). Can continuous glucose monitoring be used for the treatment of diabetes. Anal. Chem..

[73] Sekretaryova A.N., Vokhmyanina D.V., Chulanova T.O., Karyakina E.E., Karyakin A.A. (2012). Reagentless biosensor based on glucose oxidase wired by the mediator freely diffusing in enzyme containing membrane. Anal. Chem..

[74] Gough D.A., Lucisano J.Y., Tse P.H.S. (1985). Two-dimensional enzyme electrode sensor for glucose. Anal. Chem..

[75] Wang J., Lu F. (1998). Oxygen-rich oxidase enzyme electrodes for operation in oxygen-free solutions. J. Am. Chem. Soc..

[76] Wang J., Mo J.-W., Li S., Porter J. (2001). Comparison of oxygen-rich and mediator-based glucose-oxidase carbon-paste electrodes. Anal. Chim. Acta.

[77] Kontani R., Tsujimura S., Kano K. (2009). Air diffusion biocathode with CueO as electrocatalyst adsorbed on carbon particle-modified electrodes. Bioelectrochemistry.

[78] Teymourian H., Moonla C., Tehrani F., Vargas E., Aghavali R., Barfidokht A., Tangkuaram T., Mercier P.P., Dassau E., Wang J. (2019). Microneedle-based detection of ketone bodies along with glucose and lactate: Toward real-time continuous interstitial fluid monitoring of diabetic ketosis and ketoacidosis. Anal. Chem..

[79] Schuhmann W., Ohara T.J., Schmidt H.L., Heller A. (1991). Electron transfer between glucose oxidase and electrodes via redox mediators bound with flexible chains to the enzyme surface. J. Am. Chem. Soc..

[80] Ozoemena K.I., Nyokong T. (2006). Novel amperometric glucose biosensor based on an ether-linked cobalt (II) phthalocyanine–cobalt (II) tetraphenylporphyrin pentamer as a redox mediator. Electrochim. Acta.

[81] Dervisevic M., Cevik E., Şenel M. (2015). Development of glucose biosensor based on reconstitution of glucose oxidase onto polymeric redox mediator coated pencil graphite electrodes. Enzym. Microb. Technol..

[82] Al-Sagur H., Komathi S., Khan M.A., Gurek A.G., Hassan A. (2017). A novel glucose sensor using lutetium phthalocyanine as redox mediator in reduced graphene oxide conducting polymer multifunctional hydrogel. Biosens. Bioelectron..

[83] Ahmed M.U., Hossain M.M., Tamiya E. (2008). Electrochemical biosensors for medical and food applications. Electroanalysis.

[84] Teymourian H., Barfidokht A., Wang J. (2020). Electrochemical glucose sensors in diabetes management: An updated review (2010–2020). Chem. Soc. Rev..

[85] Suzuki N., Lee J., Loew N., Takahashi-Inose Y., Okuda-Shimazaki J., Kojima K., Mori K., Tsugawa W., Sode K. (2020). Engineered glucose oxidase capable of quasi-direct electron transfer after a quick-and-easy modification with a mediator. Int. J. Mol. Sci..

[86] Lee J., Hyun K., Kwon Y. (2021). A study on the stability and sensitivity of mediator-based enzymatic glucose sensor measured by catalyst consisting of multilayer stacked via layer-by-layer. J. Ind. Eng. Chem..

[87] Boussema F., Gross A.J., Hmida F., Ayed B., Majdoub H., Cosnier S., Maaref A., Holzinger M. (2018). Dawson-type polyoxometalate nanoclusters confined in a carbon nanotube matrix as efficient redox mediators for enzymatic glucose biofuel cell anodes and glucose biosensors. Biosens. Bioelectron..

[88] Marquitan M., Bobrowski T., Ernst A., Wilde P., Clausmeyer J., Ruff A., Schuhmann W. (2018). Miniaturized amperometric glucose sensors based on polymer/enzyme modified carbon electrodes in the sub-micrometer scale. J. Electrochem. Soc..

[89] Bobrowski T., Schuhmann W. (2018). Long-term implantable glucose biosensors. Curr. Opin. Electrochem..

[90] Heller A. (1992). Electrical connection of enzyme redox centers to electrodes. J. Phys. Chem..

[91] Gallay P.A., Rubianes M.D., Gutierrez F.A., Rivas G.A. (2019). Avidin and Glucose Oxidase-non-covalently Functionalized Multi-walled Carbon Nanotubes: A New Analytical Tool for Building a Bienzymatic Glucose Biosensor. Electroanalysis.

[92] Rafighi P., Tavahodi M., Haghighi B. (2016). Fabrication of a third-generation glucose biosensor using graphene-polyethyleneimine-gold nanoparticles hybrid. Sens. Actuators B Chem..

[93] Mehmeti E., Stanković D.M., Chaiyo S., Zavasnik J., Žagar K., Kalcher K. (2017). Wiring of glucose oxidase with graphene nanoribbons: An electrochemical third generation glucose biosensor. Microchim. Acta.

[94] Willner I., Heleg-Shabtai V., Blonder R., Katz E., Tao G., Bückmann A.F., Heller A. (1996). Electrical wiring of glucose oxidase by reconstitution of FAD-modified monolayers assembled onto Au-electrodes. J. Am. Chem. Soc..

[95] Fruk L., Kuo C.H., Torres E., Niemeyer C.M. (2009). Apoenzyme reconstitution as a chemical tool for structural enzymology and biotechnology. Angew. Chem. Int. Ed..

[96] Zayats M., Willner B., Willner I. (2008). Design of amperometric biosensors and biofuel cells by the reconstitution of electrically contacted enzyme electrodes. Electroanalysis.

[97] Zayats M., Katz E., Baron R., Willner I. (2005). Reconstitution of apo-glucose dehydrogenase on pyrroloquinoline quinone-functionalized Au nanoparticles yields an electrically contacted biocatalyst. J. Am. Chem. Soc..

[98] Şenel M., Nergiz C., Dervisevic M., Çevik E. (2013). Development of amperometric glucose biosensor based on reconstitution of glucose oxidase on polymeric 3-aminophenyl boronic acid monolayer. Electroanalysis.

[99] Degani Y., Heller A. (1989). Electrical communication between redox centers of glucose oxidase and electrodes via electrostatically and covalently bound redox polymers. J. Am. Chem. Soc..

[100] Yehezkeli O., Raichlin S., Tel-Vered R., Kesselman E., Danino D., Willner I. (2010). Biocatalytic implant of Pt nanoclusters into glucose oxidase: A method to electrically wire the enzyme and to transform it from an oxidase to a hydrogenase. J. Phys. Chem. Lett..

[101] Zhang H., Meng Z., Wang Q., Zheng J. (2011). A novel glucose biosensor based on direct electrochemistry of glucose oxidase incorporated in biomediated gold nanoparticles–carbon nanotubes composite film. Sens. Actuators B Chem..

[102] Tasviri M., Rafiee-Pour H.-A., Ghourchian H., Gholami M.R. (2011). Amine functionalized TiO2 coated on carbon nanotube as a nanomaterial for direct electrochemistry of glucose oxidase and glucose biosensing. J. Mol. Catal. B Enzym..

[103] Shan C., Yang H., Song J., Han D., Ivaska A., Niu L. (2009). Direct electrochemistry of glucose oxidase and biosensing for glucose based on graphene. Anal. Chem..

[104] Jose M.V., Marx S., Murata H., Koepsel R.R., Russell A.J. (2012). Direct electron transfer in a mediator-free glucose oxidase-based carbon nanotube-coated biosensor. Carbon.

[105] Holland J.T., Lau C., Brozik S., Atanassov P., Banta S. (2011). Engineering of glucose oxidase for direct electron transfer via site-specific gold nanoparticle conjugation. J. Am. Chem. Soc..

[106] Tasca F., Zafar M.N., Harreither W., Nöll G., Ludwig R., Gorton L. (2011). A third generation glucose biosensor based on cellobiose dehydrogenase from Corynascus thermophilus and single-walled carbon nanotubes. Analyst.

[107] Gupta N., Renugopalakrishnan V., Liepmann D., Paulmurugan R., Malhotra B.D. (2019). Cell-based biosensors: Recent trends, challenges and future perspectives. Biosens. Bioelectron..

[108] Bollella P., Gorton L. (2018). Enzyme based amperometric biosensors. Curr. Opin. Electrochem..

[109] Rahman M.M., Ahammad A.J.S., Jin J.-H., Ahn S.J., Lee J.-J. (2010). A Comprehensive Review of Glucose Biosensors Based on Nanostructured Metal-Oxides. Sensors.

[110] Zhu Z., Garcia-Gancedo L., Flewitt A.J., Xie H., Moussy F., Milne W.I. (2012). A Critical Review of Glucose Biosensors Based on Carbon Nanomaterials: Carbon Nanotubes and Graphene. Sensors.

[111] Wongkaew N., Simsek M., Griesche C., Baeumner A.J. (2018). Functional nanomaterials and nanostructures enhancing electrochemical biosensors and lab-on-a-chip performances: Recent progress, applications, and future perspective. Chem. Rev..

[112] Kumar-Krishnan S., García M.G.-F., Prokhorov E., Estevez-González M., Pérez R., Esparza R., Meyyappan M. (2017). Synthesis of gold nanoparticles supported on functionalized nanosilica using deep eutectic solvent for an electrochemical enzymatic glucose biosensor. J. Mater. Chem. B.

[113] Buk V., Pemble M.E. (2019). A highly sensitive glucose biosensor based on a micro disk array electrode design modified with carbon quantum dots and gold nanoparticles. Electrochim. Acta.

[114] Shrestha B.K., Ahmad R., Shrestha S., Park C.H., Kim C.S. (2017). Globular shaped polypyrrole doped well-dispersed functionalized multiwall carbon nanotubes/nafion composite for enzymatic glucose biosensor application. Sci. Rep..

[115] Li J., Liu Y., Tang X., Xu L., Min L., Xue Y., Hu X., Yang Z. (2020). Multiwalled carbon nanotubes coated with cobalt (II) sulfide nanoparticles for electrochemical sensing of glucose via direct electron transfer to glucose oxidase. Microchim. Acta.

[116] Hao L., Li S.-S., Wang J., Tan Y., Bai L., Liu A. (2020). MnO2/multi-walled carbon nanotubes based nanocomposite with enhanced electrocatalytic activity for sensitive amperometric glucose biosensing. J. Electroanal. Chem..

[117] Vukojević V., Djurdjić S., Ognjanović M., Fabian M., Samphao A., Kalcher K., Stanković D.M. (2018). Enzymatic glucose biosensor based on manganese dioxide nanoparticles decorated on graphene nanoribbons. J. Electroanal. Chem..

[118] Mao Q., Jing W., Zhou F., Liu S., Gao W., Wei Z., Jiang Z. (2021). Depositing reduced graphene oxide on ZnO nanorods to improve the performance of enzymatic glucose sensors. Mater. Sci. Semicond. Process..

[119] Hossain M.F., Slaughter G. (2020). PtNPs decorated chemically derived graphene and carbon nanotubes for sensitive and selective glucose biosensing. J. Electroanal. Chem..

[120] Kim I., Kwon D., Lee D., Lee T.H., Lee J.H., Lee G., Yoon D.S. (2018). A highly permselective electrochemical glucose sensor using red blood cell membrane. Biosens. Bioelectron..

[121] Zhao F.-Q., Keating A.F. (2007). Functional properties and genomics of glucose transporters. Curr. Genom..

[122] Manolescu A.R., Witkowska K., Kinnaird A., Cessford T., Cheeseman C. (2007). Facilitated hexose transporters: New perspectives on form and function. Physiology.

[123] Kim I., Kim C., Lee D., Lee S.W., Lee G., Yoon D.S. (2020). A bio-inspired highly selective enzymatic glucose sensor using a red blood cell membrane. Analyst.

[124] Yang Y., Zhang S., Kingston M., Jones G., Wright G., Spencer S. (2000). Glucose sensor with improved haemocompatibilty. Biosens. Bioelectron..

[125] Aydemir N., Malmström J., Travas-Sejdic J. (2016). Conducting polymer based electrochemical biosensors. Phys. Chem. Chem. Phys..

[126] Xu M., Song Y., Ye Y., Gong C., Shen Y., Wang L., Wang L. (2017). A novel flexible electrochemical glucose sensor based on gold nanoparticles/polyaniline arrays/carbon cloth electrode. Sens. Actuators B Chem..

[127] Soylemez S., Kaya H.Z., Udum Y.A., Toppare L. (2019). A multipurpose conjugated polymer: Electrochromic device and biosensor construction for glucose detection. Org. Electron..

[128] Wang L., Xu M., Xie Y., Qian C., Ma W., Wang L., Song Y. (2019). Ratiometric electrochemical glucose sensor based on electroactive Schiff base polymers. Sens. Actuators B Chem..

[129] Krishnan S.K., Prokhorov E., Bahena D., Esparza R., Meyyappan M. (2017). Chitosan-covered Pd@ Pt core–shell nanocubes for direct electron transfer in electrochemical enzymatic glucose biosensor. ACS Omega.

[130] Csoeregi E., Schmidtke D.W., Heller A. (1995). Design and optimization of a selective subcutaneously implantable glucose electrode based on “wired” glucose oxidase. Anal. Chem..

[131] Chen Y., Lu S., Zhang S., Li Y., Qu Z., Chen Y., Lu B., Wang X., Feng X. (2017). Skin-like biosensor system via electrochemical channels for noninvasive blood glucose monitoring. Sci. Adv..

[132] Arakawa T., Kuroki Y., Nitta H., Chouhan P., Toma K., Sawada S.-I., Takeuchi S., Sekita T., Akiyoshi K., Minakuchi S. (2016). Mouthguard biosensor with telemetry system for monitoring of saliva glucose: A novel cavitas sensor. Biosens. Bioelectron..

[133] Orzari L.O., de Freitas R.C., de Araujo Andreotti I.A., Gatti A., Janegitz B.C. (2019). A novel disposable self-adhesive inked paper device for electrochemical sensing of dopamine and serotonin neurotransmitters and biosensing of glucose. Biosens. Bioelectron..

[134] Nguyen T.N.H., Jin X., Nolan J.K., Xu J., Le K.V.H., Lam S., Wang Y., Alam M.A., Lee H. (2020). Printable Nonenzymatic Glucose Biosensors Using Carbon Nanotube-PtNP Nanocomposites Modified with AuRu for Improved Selectivity. ACS Biomater. Sci. Eng..

[135] Vassilyev Y.B., Khazova O.A., Nikolaeva N.N. (1985). Kinetics and mechanism of glucose electrooxidation on different electrode-catalysts: Part I. Adsorption and oxidation on platinum. J. Electroanal. Chem. Interfacial Electrochem..

[136] Vassilyev Y.B., Khazova O.A., Nikolaeva N.N. (1985). Kinetics and mechanism of glucose electrooxidation on different electrode-catalysts: Part II. Effect of the nature of the electrode and the electrooxidation mechanism. J. Electroanal. Chem. Interfacial Electrochem..

[137] Toghill K.E., Compton R.G. (2010). Electrochemical non-enzymatic glucose sensors: A perspective and an evaluation. Int. J. Electrochem. Sci..

[138] Hwang D.-W., Lee S., Seo M., Chung T.D. (2018). Recent advances in electrochemical non-enzymatic glucose sensors—A review. Anal. Chim. Acta.

[139] Yamauchi Y., Suzuki N., Radhakrishnan L., Wang L. (2009). Breakthrough and future: Nanoscale controls of compositions, morphologies, and mesochannel orientations toward advanced mesoporous materials. Chem. Rec..

[140] Mohamed M.G., Atayde E.C., Matsagar B.M., Na J., Yamauchi Y., Wu K.C.W., Kuo S.-W. (2020). Construction hierarchically mesoporous/microporous materials based on block copolymer and covalent organic framework. J. Taiwan Inst. Chem. Eng..

[141] Ci S., Huang T., Wen Z., Cui S., Mao S., Steeber D.A., Chen J. (2014). Nickel oxide hollow microsphere for non-enzyme glucose detection. Biosens. Bioelectron..

[142] Fu S., Fan G., Yang L., Li F. (2015). Non-enzymatic glucose sensor based on Au nanoparticles decorated ternary Ni-Al layered double hydroxide/single-walled carbon nanotubes/graphene nanocomposite. Electrochim. Acta.

[143] Choi T., Kim S.H., Lee C.W., Kim H., Choi S.-K., Kim S.-H., Kim E., Park J., Kim H. (2015). Synthesis of carbon nanotube–nickel nanocomposites using atomic layer deposition for high-performance non-enzymatic glucose sensing. Biosens. Bioelectron..

[144] Şavk A., Aydın H., Cellat K., Şen F. (2020). A novel high performance non-enzymatic electrochemical glucose biosensor based on activated carbon-supported Pt-Ni nanocomposite. J. Mol. Liq..

[145] Burke L.D. (1994). Premonolayer oxidation and its role in electrocatalysis. Electrochim. Acta.

[146] Norsko J.K. (1990). Chemisorption on metal surfaces. Rep. Prog. Phys..

[147] Lee S., Lee J., Park S., Boo H., Kim H.C., Chung T.D. (2018). Disposable non-enzymatic blood glucose sensing strip based on nanoporous platinum particles. Appl. Mater. Today.

[148] Verma N. (2018). A green synthetic approach for size tunable nanoporous gold nanoparticles and its glucose sensing application. Appl. Surf. Sci..

[149] Wang J., Zhao D., Xu C. (2016). Nonenzymatic electrochemical sensor for glucose based on nanoporous platinum-gold alloy. J. Nanosci. Nanotechnol..

[150] Jafarian M., Forouzandeh F., Danaee I., Gobal F., Mahjani M. (2009). Electrocatalytic oxidation of glucose on Ni and NiCu alloy modified glassy carbon electrode. J. Solid State Electrochem..

[151] Skou E. (1977). The electrochemical oxidation of glucose on platinum—I. The oxidation in 1 M H2SO4. Electrochim. Acta.

[152] Park S., Boo H., Chung T.D. (2006). Electrochemical non-enzymatic glucose sensors. Anal. Chim. Acta.

[153] Zhao Y., Li X., Schechter J.M., Yang Y. (2016). Revisiting the oxidation peak in the cathodic scan of the cyclic voltammogram of alcohol oxidation on noble metal electrodes. RSC Adv..

[154] Xu Q., Yin L., Hou C., Liu X., Hu X. (2012). Facile fabrication of nanoporous platinum by alloying–dealloying process and its application in glucose sensing. Sens. Actuators B Chem..

[155] Pasta M., La Mantia F., Cui Y. (2010). Mechanism of glucose electrochemical oxidation on gold surface. Electrochim. Acta.

[156] Hu Y., He F., Ben A., Chen C. (2014). Synthesis of hollow Pt–Ni–graphene nanostructures for nonenzymatic glucose detection. J. Electroanal. Chem..

[157] Unmüssig T., Weltin A., Urban S., Daubinger P., Urban G.A., Kieninger J. (2018). Non-enzymatic glucose sensing based on hierarchical platinum micro-/nanostructures. J. Electroanal. Chem..

[158] Weremfo A., Fong S.T.C., Khan A., Hibbert D.B., Zhao C. (2017). Electrochemically roughened nanoporous platinum electrodes for non-enzymatic glucose sensors. Electrochim. Acta.

[159] Hu Y., Niu X., Zhao H., Tang J., Lan M. (2015). Enzyme-free amperometric detection of glucose on platinum-replaced porous copper frameworks. Electrochim. Acta.

[160] Lin K.-C., Yang C.-Y., Chen S.-M. (2015). Fabrication of a nonenzymatic glucose sensor based on multi-walled carbon nanotubes decorated with platinum and silver hybrid composite. Int. J. Electrochem. Sci.

[161] Xiao F., Li Y., Gao H., Ge S., Duan H. (2013). Growth of coral-like PtAu–MnO2 binary nanocomposites on free-standing graphene paper for flexible nonenzymatic glucose sensors. Biosens. Bioelectron..

[162] Zhang C., Zhang R., Gao X., Cheng C., Hou L., Li X., Chen W. (2018). Small naked Pt nanoparticles confined in mesoporous shell of hollow carbon spheres for high-performance nonenzymatic sensing of H_2_O_2_ and glucose. ACS Omega.

[163] Chang G., Shu H., Huang Q., Oyama M., Ji K., Liu X., He Y. (2015). Synthesis of highly dispersed Pt nanoclusters anchored graphene composites and their application for non-enzymatic glucose sensing. Electrochim. Acta.

[164] Li C., Iqbal M., Lin J., Luo X., Jiang B., Malgras V., Wu K.C.W., Kim J., Yamauchi Y. (2018). Electrochemical deposition: An advanced approach for templated synthesis of nanoporous metal architectures. Acc. Chem. Res..

[165] Kim S.H., Choi J.B., Nguyen Q.N., Lee J.M., Park S., Chung T.D., Byun J.Y. (2013). Nanoporous platinum thin films synthesized by electrochemical dealloying for nonenzymatic glucose detection. Phys. Chem. Chem. Phys..

[166] Park S., Chung T.D., Kim H.C. (2003). Nonenzymatic glucose detection using mesoporous platinum. Anal. Chem..

[167] Burke L., Ryan T. (1992). The role of incipient hydrous oxides in the oxidation of glucose and some of its derivatives in aqueous media. Electrochim. Acta.

[168] Pasta M., Ruffo R., Falletta E., Mari C., Della Pina C. (2010). Alkaline glucose oxidation on nanostructured gold electrodes. Gold Bull..

[169] Xue Y., Wang S., Shi P., Huang Y., Scaglione F., Rizzi P., Battezzati L., Denis P., Fecht H.-J. (2019). Nanoporous gold chemically de-alloyed from Au-based amorphous thin film for electrochemical nonenzymatic H2O2 sensing. Chem. Phys. Lett..

[170] Chen A., Wang J., Wang Y., Jia Y., Gu J., Xie X., Pan D. (2015). Effects of pore size and residual Ag on electrocatalytic properties of nanoporous gold films prepared by pulse electrochemical dealloying. Electrochim. Acta.

[171] Bai Y., Yang W., Sun Y., Sun C. (2008). Enzyme-free glucose sensor based on a three-dimensional gold film electrode. Sens. Actuators B Chem..

[172] Cherevko S., Chung C.-H. (2009). Gold nanowire array electrode for non-enzymatic voltammetric and amperometric glucose detection. Sens. Actuators B Chem..

[173] Sanzó G., Taurino I., Antiochia R., Gorton L., Favero G., Mazzei F., De Micheli G., Carrara S. (2016). Bubble electrodeposition of gold porous nanocorals for the enzymatic and non-enzymatic detection of glucose. Bioelectrochemistry.

[174] Han L., Zhang S., Han L., Yang D.-P., Hou C., Liu A. (2014). Porous gold cluster film prepared from Au@ BSA microspheres for electrochemical nonenzymatic glucose sensor. Electrochim. Acta.

[175] Scandurra A., Ruffino F., Sanzaro S., Grimaldi M.G. (2019). Laser and thermal dewetting of gold layer onto graphene paper for non-enzymatic electrochemical detection of glucose and fructose. Sens. Actuators B Chem..

[176] Niu X., Li X., Pan J., He Y., Qiu F., Yan Y. (2016). Recent advances in non-enzymatic electrochemical glucose sensors based on non-precious transition metal materials: Opportunities and challenges. RSC Adv..

[177] Tee S.Y., Teng C.P., Ye E. (2017). Metal nanostructures for non-enzymatic glucose sensing. Mater. Sci. Eng. C.

[178] Berchmans S., Gomathi H., Rao G.P. (1995). Electrooxidation of alcohols and sugars catalysed on a nickel oxide modified glassy carbon electrode. J. Electroanal. Chem..

[179] Fleischmann M., Korinek K., Pletcher D. (1971). The oxidation of organic compounds at a nickel anode in alkaline solution. J. Electroanal. Chem. Interfacial Electrochem..

[180] Marioli J.M., Kuwana T. (1992). Electrochemical characterization of carbohydrate oxidation at copper electrodes. Electrochim. Acta.

[181] Kano K., Torimura M., Esaka Y., Goto M., Ueda T. (1994). Electrocatalytic oxidation of carbohydrates at copper (II)-modified electrodes and its application to flow-through detection. J. Electroanal. Chem..

[182] Perušković D.S., Stevanović N.R., Kovačević G.N., Stanković D.M., Lolić A.Đ., Baošić R.M. (2020). Application of N, N’-Bis (acetylacetonato) propylenediimine Copper (II) Complex as Mediator for Glucose Biosensor. Chem. Select.

[183] Niu X., Li Y., Tang J., Hu Y., Zhao H., Lan M. (2014). Electrochemical sensing interfaces with tunable porosity for nonenzymatic glucose detection: A Cu foam case. Biosens. Bioelectron..

[184] Riman D., Spyrou K., Karantzalis A.E., Hrbac J., Prodromidis M.I. (2017). Glucose sensing on graphite screen-printed electrode modified by sparking of copper nickel alloys. Talanta.

[185] Chen X., Tian X., Zhao L., Huang Z., Oyama M. (2014). Nonenzymatic sensing of glucose at neutral pH values using a glassy carbon electrode modified with graphene nanosheets and Pt-Pd bimetallic nanocubes. Microchim. Acta.

[186] Chen H., Fan G., Zhao J., Qiu M., Sun P., Fu Y., Han D., Cui G. (2019). A portable micro glucose sensor based on copper-based nanocomposite structure. New J. Chem..

[187] Zhang Y., Li N., Xiang Y., Wang D., Zhang P., Wang Y., Lu S., Xu R., Zhao J. (2020). A flexible non-enzymatic glucose sensor based on copper nanoparticles anchored on laser-induced graphene. Carbon.

[188] Liu X., Yang W., Chen L., Jia J. (2017). Three-dimensional copper foam supported CuO nanowire arrays: An efficient non-enzymatic glucose sensor. Electrochim. Acta.

[189] Gao X., Feng W., Zhu Z., Wu Z., Li S., Kan S., Qiu X., Guo A., Chen W., Yin K. (2021). Rapid Fabrication of Superhydrophilic Micro/Nanostructured Nickel Foam Toward High-Performance Glucose Sensor. Adv. Mater. Interfaces.

[190] Wang F., Chen X., Chen L., Yang J., Wang Q. (2019). High-performance non-enzymatic glucose sensor by hierarchical flower-like nickel (II)-based MOF/carbon nanotubes composite. Mater. Sci. Eng. C.

[191] Zhang X., Xu Y., Ye B. (2018). An efficient electrochemical glucose sensor based on porous nickel-based metal organic framework/carbon nanotubes composite (Ni-MOF/CNTs). J. Alloy. Compd..

[192] Aoun S.B., Dursun Z., Koga T., Bang G.S., Sotomura T., Taniguchi I. (2004). Effect of metal ad-layers on Au (1 1 1) electrodes on electrocatalytic oxidation of glucose in an alkaline solution. J. Electroanal. Chem..

[193] Zhao Y., Fan L., Hong B., Ren J., Zhang M., Que Q., Ji J. (2016). Nonenzymatic detection of glucose using three-dimensional PtNi nanoclusters electrodeposited on the multiwalled carbon nanotubes. Sens. Actuators B Chem..

[194] Xiao F., Zhao F., Mei D., Mo Z., Zeng B. (2009). Nonenzymatic glucose sensor based on ultrasonic-electrodeposition of bimetallic PtM (M = Ru, Pd and Au) nanoparticles on carbon nanotubes–ionic liquid composite film. Biosens. Bioelectron..

[195] Li J., Koinkar P., Fuchiwaki Y., Yasuzawa M. (2016). A fine pointed glucose oxidase immobilized electrode for low-invasive amperometric glucose monitoring. Biosens. Bioelectron..

[196] Guascito M.R., Chirizzi D., Malitesta C., Siciliano M., Siciliano T., Tepore A. (2012). Amperometric non-enzymatic bimetallic glucose sensor based on platinum tellurium microtubes modified electrode. Electrochem. Commun..

[197] Yang Z., Miao Y., Xu L., Song G., Zhou S. (2015). Adsorption of Bi III on Pt nanoparticles leading to the enhanced electrocatalysis of glucose oxidation. Colloid J..

[198] Nantaphol S., Watanabe T., Nomura N., Siangproh W., Chailapakul O., Einaga Y. (2017). Bimetallic Pt–Au nanocatalysts electrochemically deposited on boron-doped diamond electrodes for nonenzymatic glucose detection. Biosens. Bioelectron..

[199] Guo T., Zhang Y., Ouyang Y., Yu G., Liao Y., Zhang Z. (2016). Facile Synthesis of Bimetallic PtxCu1-x Nanostrands and Their Application in Non-Enzymatic Glucose Sensor. Int. J. Electrochem. Sci..

[200] Pandey P.C., Panday D., Pandey G. (2014). 3-Aminopropyltrimethoxysilane and organic electron donors mediated synthesis of functional amphiphilic gold nanoparticles and their bioanalytical applications. RSC Adv..

[201] Marketsandmarkets Biosensors Market. https://www.marketsandmarkets.com/Market-Reports/biosensors-market-798.html.

[202] Abrera A.T., Sützl L., Haltrich D. (2020). Pyranose oxidase: A versatile sugar oxidoreductase for bioelectrochemical applications. Bioelectrochemistry.

[203] Brugger D., Krondorfer I., Shelswell C., Huber-Dittes B., Haltrich D., Peterbauer C.K. (2014). Engineering pyranose 2-oxidase for modified oxygen reactivity. PLoS ONE.

[204] Kurbanoglu S., Zafar M.N., Tasca F., Aslam I., Spadiut O., Leech D., Haltrich D., Gorton L. (2018). Amperometric Flow Injection Analysis of Glucose and Galactose Based on Engineered Pyranose 2-Oxidases and Osmium Polymers for Biosensor Applications. Electroanalysis.

[205] Zafar M.Q., Zhao H. (2019). 4D Printing: Future Insight in Additive Manufacturing. Met. Mater. Int..

[206] Zhang Z., Demir K.G., Gu G.X. (2019). Developments in 4D-printing: A review on current smart materials, technologies, and applications. Int. J. Smart Nano Mater..

[207] Maniruzzaman M. (2019). 3D and 4D Printing in Biomedical Applications: Process Engineering and Additive Manufacturing.

[208] Benson J., Fung C.M., Lloyd J.S., Deganello D., Smith N.A., Teng K.S. (2015). Direct patterning of gold nanoparticles using flexographic printing for biosensing applications. Nanoscale Res. Lett..

[209] Setti L., Fraleoni-Morgera A., Ballarin B., Filippini A., Frascaro D., Piana C. (2005). An amperometric glucose biosensor prototype fabricated by thermal inkjet printing. Biosens. Bioelectron..

[210] Basnar B., Willner I. (2009). Dip-pen-nanolithographic patterning of metallic, semiconductor, and metal oxide nanostructures on surfaces. Small.

[211] Taleat Z., Khoshroo A., Mazloum-Ardakani M. (2014). Screen-printed electrodes for biosensing: A review (2008–2013). Microchim. Acta.

[212] Saunders R.E., Derby B. (2014). Inkjet printing biomaterials for tissue engineering: Bioprinting. Int. Mater. Rev..

[213] Salaita K., Wang Y., Mirkin C.A. (2007). Applications of dip-pen nanolithography. Nat. Nanotechnol..

[214] Park S., Park S., Jeong R.-A., Boo H., Park J., Kim H.C., Chung T.D. (2012). Nonenzymatic continuous glucose monitoring in human whole blood using electrified nanoporous Pt. Biosens. Bioelectron..

[215] Duda T., Raghavan L.V. (2016). 3D metal printing technology. IFAC Pap..

[216] Rana J., Jindal J., Beniwal V., Chhokar V. (2010). Utility biosensors for applications in agriculture—A Review. J. Am. Sci..

[217] Otero F., Magner E. (2020). Biosensors—recent advances and future challenges in electrode materials. Sensors.

